# Katanin-like 2 (KATNAL2) functions in multiple aspects of haploid male germ cell development in the mouse

**DOI:** 10.1371/journal.pgen.1007078

**Published:** 2017-11-14

**Authors:** Jessica E. M. Dunleavy, Hidenobu Okuda, Anne E. O’Connor, D. Jo Merriner, Liza O’Donnell, Duangporn Jamsai, Martin Bergmann, Moira K. O’Bryan

**Affiliations:** 1 Development and Stem Cells Program, Monash Biomedicine Discovery Institute and The Department of Anatomy and Developmental Biology, Monash University, Melbourne, Victoria; Australia; 2 School of Biological Sciences, Monash University, Melbourne, Victoria; Australia; 3 Hudson Institute of Medical Research and Department of Molecular and Translational Science, Monash University, Melbourne, Victoria; Australia; 4 Institute of Veterinary Anatomy, Histology and Embryology, Justus Liebig University Giessen, Giessen, Hesse; Germany; Baylor College of Medicine, UNITED STATES

## Abstract

The katanin microtubule-severing proteins are essential regulators of microtubule dynamics in a diverse range of species. Here we have defined critical roles for the poorly characterised katanin protein KATNAL2 in multiple aspects of spermatogenesis: the initiation of sperm tail growth from the basal body, sperm head shaping via the manchette, acrosome attachment, and ultimately sperm release. We present data suggesting that depending on context, KATNAL2 can partner with the regulatory protein KATNB1 or act autonomously. Moreover, our data indicate KATNAL2 may regulate δ- and ε-tubulin rather than classical α-β-tubulin microtubule polymers, suggesting the katanin family has a greater diversity of function than previously realised. Together with our previous research, showing the essential requirement of katanin proteins KATNAL1 and KATNB1 during spermatogenesis, our data supports the concept that in higher order species the presence of multiple katanins has allowed for subspecialisation of function within complex cellular settings such as the seminiferous epithelium.

## Author summary

Male infertility affects one in twenty men of reproductive age in western countries. Despite this, the biochemical basis of common defects, including reduced sperm count and abnormal sperm structure and function, remains poorly defined. Microtubules are cellular “scaffolds” that serve critical roles in all cells, including developing male germ cells wherein they facilitate mitosis and meiosis (cell division), sperm head remodelling and sperm tail formation. The precise regulation of microtubule number, length and movement is thus, essential for male fertility. Within this manuscript, we have used spermatogenesis to define the function of the putative microtubule-severing protein katanin-like 2 (KATNAL2). We show that mice with compromised KATNAL2 function are male sterile as a consequence of defects in the structural remodelling of germ cells. Notably, we show the function of microtubule-based structures involved in sperm head shaping and tail formation are disrupted. Further, we show for the first time, that KATNAL2 can function both independently or in concert with the katanin regulatory protein KATNB1 and that it can target the poorly characterized tubulin subunits delta and epsilon. Our research has immediate relevance to the origins of human male infertility and provides novel insights into aspects of microtubule regulation relevant to numerous tissues and species.

## Introduction

The katanins are members of the ATPases Associated with diverse cellular Activities (AAA) superfamily, and were first identified via the microtubule severing activity of the catalytic KATNA1 (p60) and its regulatory protein, KATNB1 (p80) [1], and their pivotal roles in defining meiotic spindle structure in *Caenorhabditis elegans* [2,3]. Since then, the KATNA1-KATNB1 complex has emerged as a critical regulator of microtubule dynamics in a range of contexts, including mitosis, cilia biogenesis and disassembly, neurogenesis and cell migration [4,5].

In its active ATP-bound state, KATNA1 forms hexameric rings capable of binding to and severing microtubule polymers [1,6–8]. Typically, KATNA1 binding to KATNB1 enhances severing, likely due to KATNB1 increasing the stability of the KATNA1 hexamer [6,9,10]. Although intrinsically destructive, microtubule severing is also used to remodel existing structures, release microtubules from nucleation sites and to generate short stable microtubule fragments that can ‘seed’ new growth and/or be easily transported around the cell [11–14]. Reflective of their integral role in microtubule dynamics, *Katna1* and *Katnb1* are highly conserved across the genomes of animals, higher order plants and protozoa. In a number of higher order species, two paralogues of *Katna1*, namely *Katnal1* and *Katnal2*, also exist [5]. KATNAL1 is required for sensory neuron dendrite arborisation and pruning in *Drosophila melanogaster* [15,16] and is capable of being regulated by KATNB1 [17]. In comparison, KATNAL2 is poorly characterised. KATNAL2 has been proposed as a risk factor for human autism [18–20] and viral transfection studies suggest a role in dendrite arborisation in developing mouse neurons [21]. *In vitro* studies have pointed to functions in centriole dynamics and ciliogenesis [17,22]. An *in vivo* role for KATNAL2 remains untested.

Mammalian spermatogenesis is exquisitely sensitive to disturbances in microtubules. The microtubule cytoskeleton provides an essential and dynamic scaffold that drives many of the structural changes in mitosis, meiosis and spermatid remodelling (spermiogenesis), and the complex interactions between developing germ cells and their supporting Sertoli cells [23]. Recently, we have shown that multiple aspects of microtubule function in the adult male germ line depend on the action of KATNB1, including meiotic spindle structure and cytokinesis, axoneme development and thus sperm motility, and sperm head shaping [24]. The precise severing proteins mediating each of these phenotypes however, remain to be defined. Each of the three KATNA1-related subunits is expressed in the seminiferous epithelium [24] and towards an understanding of the function of each within male fertility, we have shown that KATNAL1 is required for Sertoli cell function, specifically in defining germ cell positioning within the depth of the epithelium and maintaining Sertoli-round spermatid adhesion [25].

Here we report that KATNAL2 mediates many of the post-meiotic aspects of KATNB1 function, including sperm head shaping. We provide additional evidence that KATNAL2 is capable of acting in a KATNB1-independent manner, including in basal body extension and spermiation, and that KATNAL2 has the potential to interact with the poorly characterized tubulin sub-types δ and ε. Collectively, these data paint an emerging picture of katanin sub-specialisation to ensure the appropriate development of multiple microtubule-dependent structures during male germ cell development.

## Results

### KATNAL2 is highly enriched in the testis wherein multiple isoforms are produced

**Fig 1:**
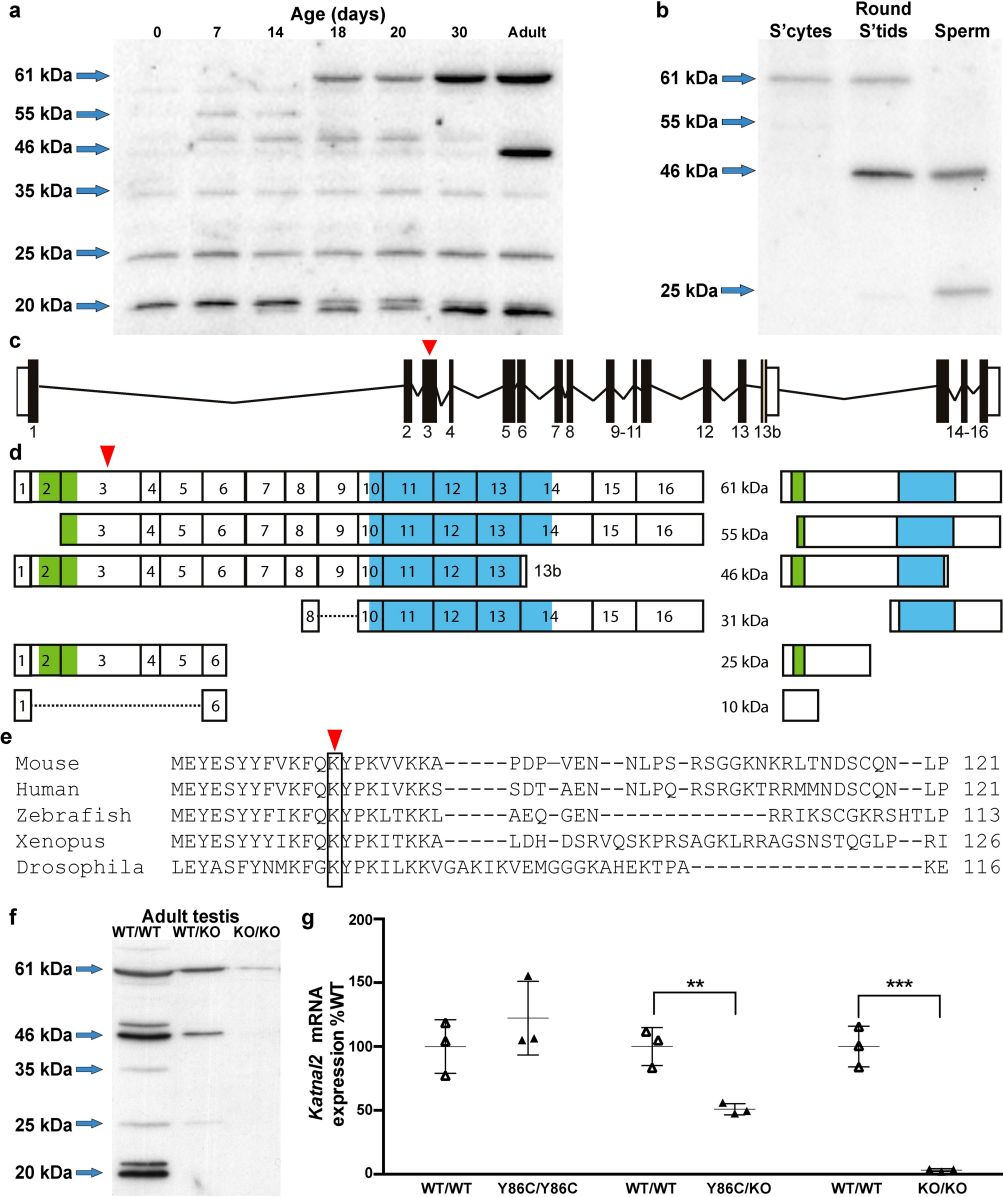
Multiple KATNAL2 isoforms are produced and differentially expressed in the testis. (**a**) Western blot analysis of KATNAL2 protein expression in whole testis homogenates from *Katnal2*^WT/WT^ mice of various ages. (**b**) Western blot analysis of KATNAL2 protein expression in isolated germ cell populations from adult *Katnal2*^WT/WT^ mice. S’cytes = spermatocytes, round s’tids = round spermatids. Schematic representation of the *Katnal2* gene (**c**) and the various predicted transcripts and their corresponding proteins (**d**). Location of the LisH and AAA ATPase domains are shown in green and blue respectively. (**e**) A cross-phyla comparison of the region containing the Y86C mutation. Red arrowheads indicate the position of the Y86C mutation. (**f**) Western blot analysis of KATNAL2 protein in whole testis homogenates from *Katnal2*^WT/WT^, *Katnal2*^WT/KO^ and *Katnal2*^KO/KO^ mice. (**g**) qPCR analysis of *Katnal2* transcript levels in whole testis homogenates from *Katnal2*^Y86C/Y86C^, *Katnal2*^Y86C/KO^ and *Katnal2*^KO/KO^ mice relative to *Katnal2*^WT/WT^ (n = 3/group). No difference was observed in *Katnal2* transcript levels within the testis between *Katnal2*^Y86C/Y86C^ and *Katnal2*^WT/WT^ mice. However, *Katnal2* mRNA transcript levels in the testis were reduced by 49% and 97% in *Katnal2*^WT/KO^ and *Katnal2*^KO/KO^ mice respectively, compared to *Katnal2*^WT/WT^. Lines represent mean± SD, ** p<0.01 compared to *Katnal2*^WT/WT^, *** p<0.001 compared to *Katnal2*^WT/WT^.

Previously we have shown that *Katnal2* is highly testis-enriched [24]. To refine this analysis, we took testes from mice at defined ages during the establishment of spermatogenesis and assessed them by western blotting for KATNAL2 content. As the establishment of spermatogenesis, and thus germ cell content, is tightly regulated in the mouse, age-dependent changes in gene expression can indicate the cell of origin. Total testis expression was compared to expression in isolated germ cell populations. The antisera used was designed to bind to the maximum number of predicted KATNAL2 isoforms as listed in Ensembl, but not cross-react with other katanin-like proteins. As shown in Fig 1A and 1B, several KATNAL2 isoforms were notably enriched in particular ages and cell types. Collectively, these results indicate that the 55kDa isoform was enriched within spermatogonia, which appear at post-natal day 6, then became progressively diluted as the testis was populated with more mature germ cells types. The 61kDa isoform was enriched in spermatocytes and spermatids, but excluded from mature sperm. The 46kDa isoform was only seen in testes samples >30 days of age and in mature sperm, raising the possibility of its expression in elongated spermatids. By contrast, the 20 and 35kDa isoforms were constitutively produced in the testis, but were not detected in germ cells, suggesting they are produced in Sertoli cells and/or other somatic cells. The 25kDa isoform was also constitutively produced in the testis at all ages and was present in mature sperm. The molecular weight of the 61kDa, 55kDa, 46kDa and 25kDa isoforms was consistent with four of the six isoforms predicted in Ensembl (Fig 1D). The 31 and 10 kDa isoforms predicted in Ensembl would not be detected by our antibody as they lack the amino acids encoded by exon three against which our antibody was raised (Fig 1D). In addition, we detected 35 and 20kDa proteins, suggesting that additional exon 3 containing isoforms are encoded within the testis, but are yet to be recognized by Ensembl. The specificity of the KATNAL2 antibody was confirmed using western blotting (Fig 1F) and immunochemistry (S1 Fig).

Consistent with the western blotting data, immunochemistry data indicated that KATNAL2 was localised in highest concentrations in elongating and elongated spermatids, where it is concentrated around the spermatid head (S1 Fig).

### KATNAL2 plays an essential role in sperm head shaping, axoneme initiation and sperm release

In order to test the *in vivo* function of KATNAL2, mice containing a tyrosine (TAC) to cysteine (TGC) substitution in exon 3 of the *Katnal2* gene (Fig 1C–1E) were obtained from the missense mutation library of the Australian Phenomics Network. Tyrosine 86 is conserved in all species within which KATNAL2 orthologues are observed (Fig 1E). Following at least two rounds of breeding with non-mutated mice, heterozygous mutant mice were inter-crossed and the resultant wild type and *Katnal2*^*Y86C/Y86C*^ offspring test mated with wild type females. *Katnal2*^*Y86C/Y86C*^ animals appeared outwardly normal, had a normal mating frequency, but were sterile (7.5 pups per copulatory plug in *Katnal2*^*WT/WT*^ (n = 3) vs 0.00 in *Katnal2*^*Y86C/Y86C*^ (n = 9) p = <0.0001).

To confirm the essential nature of KATNAL2 for male fertility, we produced *Katnal2* knockout (*Katnal2*^*KO/KO*^) mice using knockout first conditional-ready embryonic stem cells produced by the International Mouse Knockout Consortium (S3 Fig). Both *Katnal2*^*KO/KO*^ (S3 Fig) and *Katnal2*^*Y86C/KO*^ (S2 Fig) males were sterile and contained an apparently identical male fertility phenotype to that observed in *Katnal2*^*Y86C/Y86C*^ mice, thus confirming the essential requirement for KATNAL2 in spermatogenesis and that the Y86C allele can be considered a null (or very close to null) allele. As expected, *Katnal2*^*KO/KO*^ produced virtually no *Katnal2* mRNA (Fig 1G) and KATNAL2 protein (Fig 1F). By contrast, *Katnal2*^*Y86C/Y86C*^ testes contained comparable amounts of *Katnal2* mRNA to wild type littermates (Fig 1G) but 41% of wild type KATNAL2 levels (S2 Fig). As expected, testes from *Katnal2*^*Y86C/KO*^ mice contained *Katnal2* mRNA levels intermediate between *Katnal2*^*Y86C/Y86C*^ and *Katnal2*^*KO/KO*^ (Fig 1G). These data suggest that tyrosine 86 has an essential role in KATNAL2 function and stability. We cannot, however, rule out the possibility that the introduction of an additional cysteine in the Y86C mutants resulted in a catastrophic change in protein conformation. Collectively, these data suggest that the *Katnal2*^*Y86C/Y86C*^ phenotype was not due to a gain of function i.e. it is equivalent to the *Katnal2*^*KO/KO*^ phenotype. For simplicity, the bulk of data presented in the body of the paper is from *Katnal2*^*Y86C/Y86C*^ mice, with confirmation from *Katnal2*^*KO/KO*^ and *Katnal2*^*Y86C/KO*^ contained in the supplemental data.

**Fig 2:**
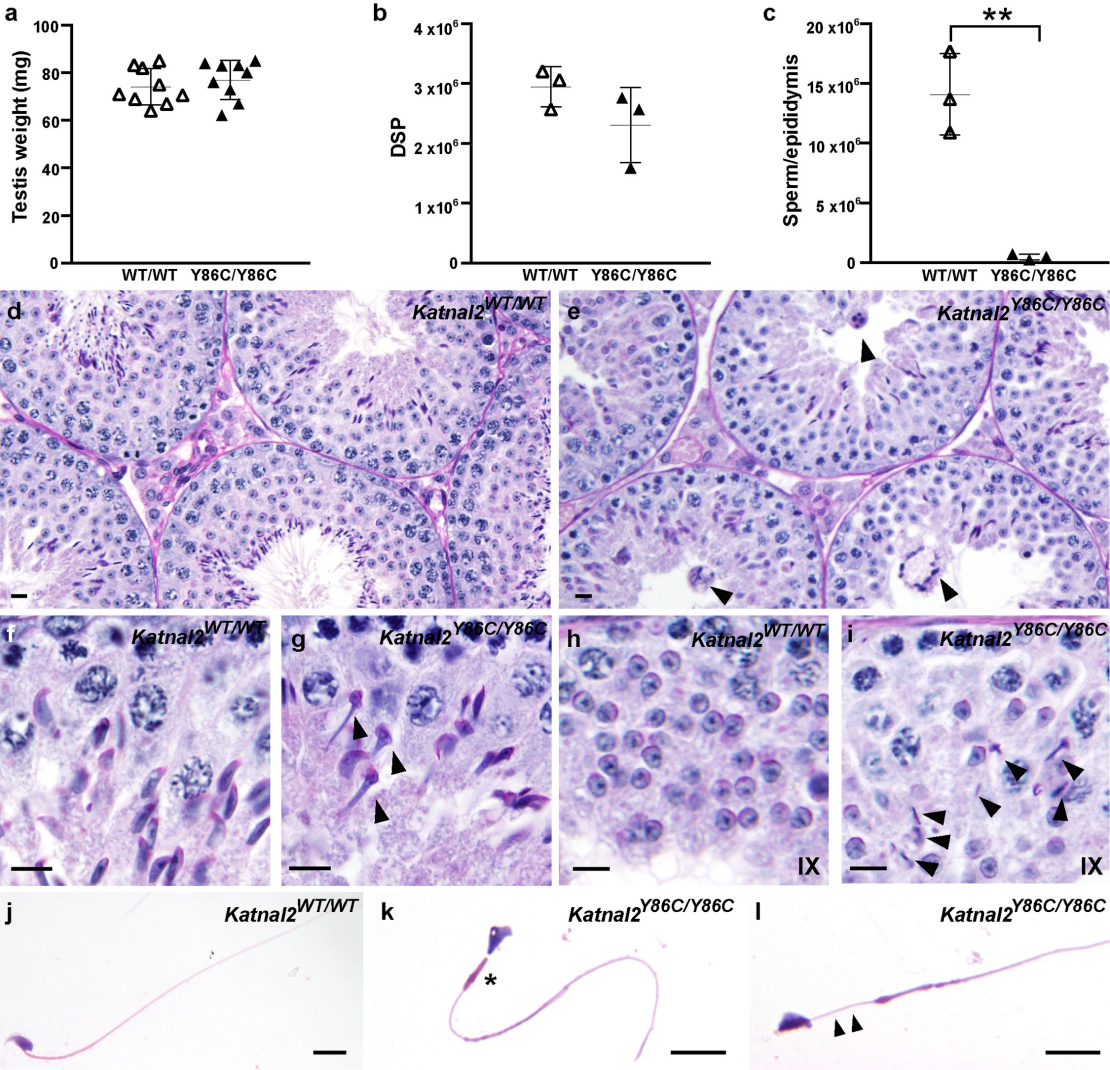
Spermatogenic defects in *Katnal2*^Y86C/Y86C^ mice. (**a**). Testis weight in *Katnal2*^WT/WT^ (white triangles) and *Katnal2*^Y86C/Y86C^ (black triangles) mice (n = 9/group). Daily sperm production (DSP) in the testes (**b**) and total epididymal sperm content (**c**) of *Katnal2*^WT/WT^ (white triangles) and *Katnal2*^Y86C/Y86C^ (black triangles) mice (n = 3/group). Total epididymal sperm content was reduced by 96.5% in *Katnal2*^Y86C/Y86C^ compared to *Katnal2*^WT/WT^. In **a**–**c**, lines represent mean ± SD, ** p<0.01 compared to *Katnal2*^WT/WT^. (**d**–**i)** Periodic acid Schiff’s (PAS) stained testis sections from *Katnal2*^WT/WT^ and *Katnal2*^Y86C/Y86C^ mice. Genotype is indicated in the top right-hand corner. Multinucleated symplasts (arrowheads) were frequently observed in the *Katnal2*^Y86C/Y86C^ seminiferous epithelium (**e**). (**f**–**g**) Elongating spermatids in *Katnal2*^WT/WT^ versus *Katnal2*^Y86C/Y86C^ mice. Abnormal nuclear (club-shaped) morphology of spermatids (arrowheads) was frequently observed in *Katnal2*^Y86C/Y86C^ mice. **(h**–**i)** Spermiation in *Katnal2*^WT/WT^ versus *Katnal2*^Y86C/Y86C^ mice. At the cessation of spermatogenesis, retained elongated spermatids (arrowheads) were frequently observed in stage IX tubules of *Katnal2*^Y86C/Y86C^ (**i**) but were rarely observed in *Katnal2*^WT/WT^ (**h**) mice. **(j**–**l)** Cauda epididymal sperm morphology in *Katnal2*^WT/WT^ and *Katnal2*^Y86C/Y86C^ mice. *Katnal2*^Y86C/Y86C^ sperm frequently displayed abnormal (asterisk) or absent (arrowheads) mitochondrial mid-pieces. Sperm heads were often abnormally shaped. Scale bars in **d**–**l** = 10μm.

A histological assessment of the male reproductive tract quickly identified that the origin of sterility in *Katnal2*^*Y86C/Y86C*^ males was the complete absence of motile sperm within the epididymides (Fig 2). Both testis weight and daily sperm production were normal in *Katnal2*^*Y86C/Y86C*^ males (Fig 2A and 2B), as were the numbers of apoptotic germ cells (S6 Fig). Epididymal sperm content was, however, reduced by 96.5% compared to that seen in wild type littermates (Fig 2C), and, of the few sperm that were found within the epididymis, all possessed abnormal sperm head shape, short or absent tails, an abnormally formed mitochondrial sheath in their mid-piece (Fig 2J–2L) and had no capacity for motility. The dramatic difference in the ratio of daily sperm production and epididymal sperm content in wild type versus *Katnal2*^*Y86C/Y86C*^ males is indicative of a massive failure of spermiation. This failure was easily seen in stage IX tubules of *Katnal2*^*Y86C/Y86C*^ males wherein elongated spermatids were frequently observed within the depth of the seminiferous epithelium prior to their destruction by Sertoli cells (Fig 2I). By contrast, elongated spermatids were rarely observed in stage IX tubules in wild type littermates (Fig 2H). The origin of this phenotype is described in more detail below and it was also seen in *Katnal2*^*KO/KO*^ and *Katnal2*^*Y86C/KO*^ males (S2 and S3 Figs).

A close examination of the testis revealed that the bulk of the abnormalities observed in *Katnal2*^*Y86C/Y86C*^ males arose during spermiogenesis (haploid germ cell development) (Fig 2). Testis histology was relatively normal in pre-meiotic and meiotic germ cells. As meiotic abnormalities were abundant in the KATNB1 hypomorphic mice (*Katnb1*^*Taily/Taily*^)[24], these data suggest that KATNB1 function in male meiosis was not achieved in partnership with KATNAL2.

**Fig 3:**
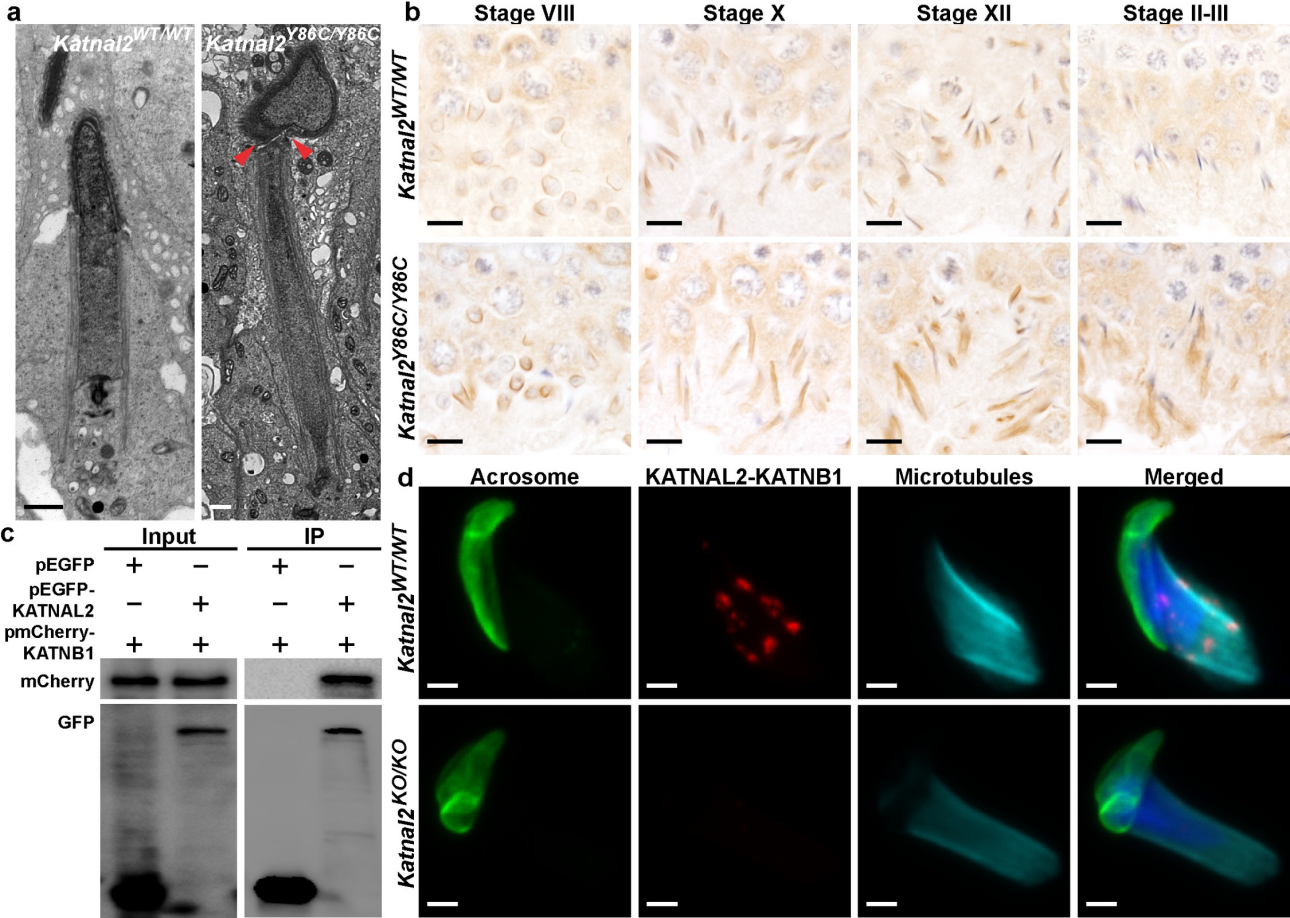
KATNAL2 regulates the manchette, and thus spermatid head shaping, in association with KATNB1. **(a)** Electron microscopy showing nuclear morphology of elongating spermatids in *Katnal2*^WT/WT^ and *Katnal2*^Y86C/Y86C^ mice. Excessive constriction of the perinuclear ring (arrowheads) was observed in *Katnal2*^Y86C/Y86C^ but not *Katnal2*^WT/WT^ mice. Scale bars in **a** = 2 μm. **(b)** α-tubulin immunolabelling as a marker for spermatid manchettes in *Katnal2*^WT/WT^ and *Katnal2*^Y86C/Y86C^ testis sections. Primary antibody negative controls are included in S7 Fig Scale bars in **b** = 10 μm. **(c)** KATNAL2 interaction with KATNB1 was indicated by co-immunoprecipitation of the pEGFP-KATNAL2 –pmCherry-KATNB1 complex using anti-GFP beads and confirmed by an *in situ* proximity ligation assay (**d**). (**c**) Input: whole cell lysate from transfected cells. IP: immunoprecipitation with GFP conjugated beads. The left upper panel shows mCherry-KATNB1 was successfully transfected into both cell populations and the left lower panel shows EGFP and EGFP-KATNAL2 are successfully transfected into the appropriate cell populations. The right upper panel confirmed mCherry-KATNB1 can bind to EGFP-KATNAL2, but not to EGFP. Right lower panel confirmed EGFP and EGFP-KATNAL2 proteins were successfully precipitated with GFP beads. **(d)** Representative images of an *in situ* proximity ligation assay using antibodies directed against KATNB1 and KATNAL2 in isolated *Katnal2*^WT/WT^ and *Katnal2*^KO/KO^ elongating spermatids. Cells were counterstained with DAPI to visualize DNA, with PNA to visualize the acrosome and α-tubulin immunolabeled as a marker of microtubules. Manufacturer recommended negative controls are included in S8 Fig Scale bars in **d** = 2 μm.

**Fig 4:**
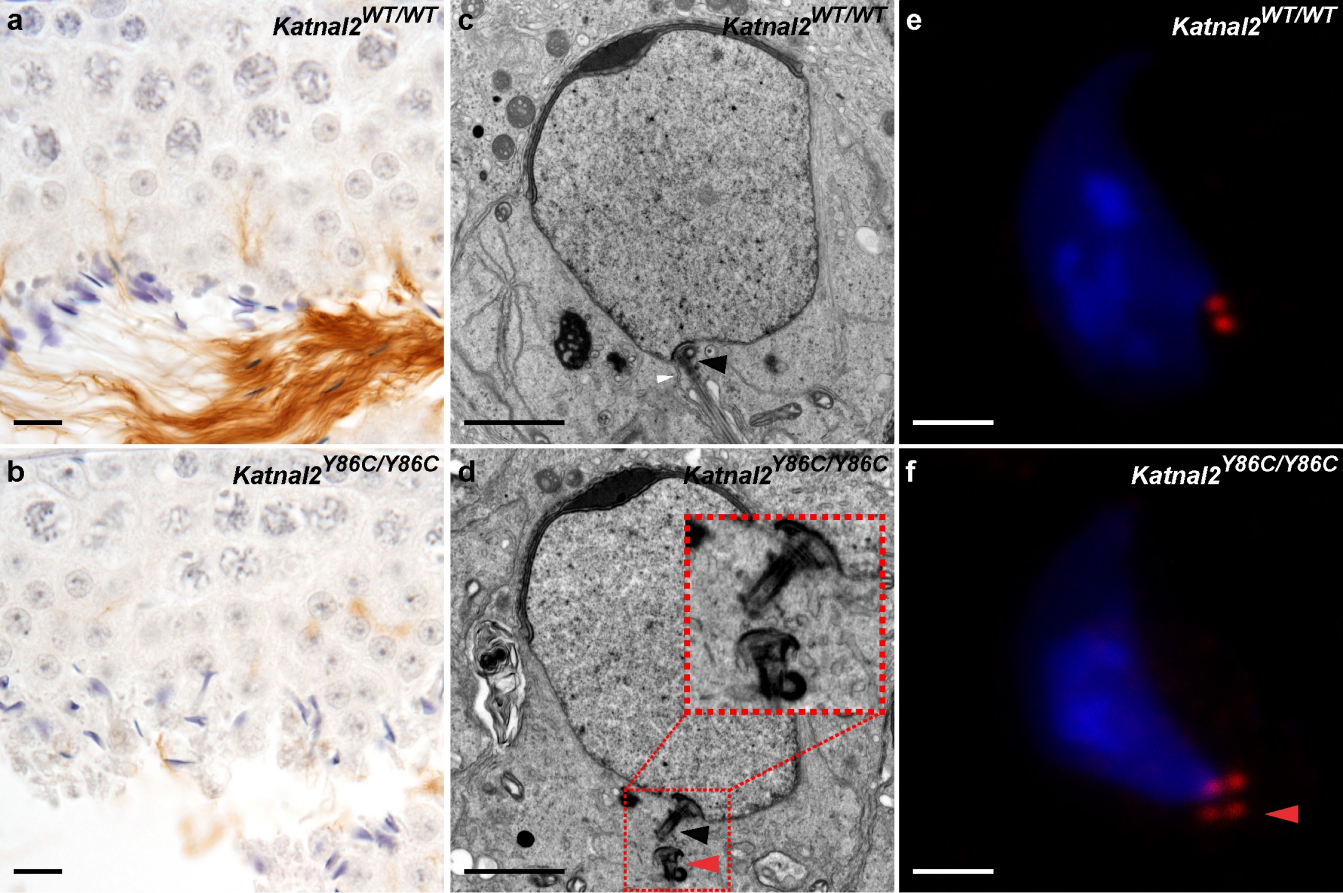
KATNAL2 has essential roles in tail formation and centriole number regulation. Acetylated tubulin immunolabelling as a marker for sperm tails in *Katnal2*^WT/WT^ (**a**) and *Katnal2*^Y86C/Y86C^ (**b**) testis sections. Sperm tail content was markedly reduced in *Katnal2*^Y86C/Y86C^ versus *Katnal2*^WT/WT^. Scale bars in **a**–**b** = 10 μm. In both *Katnal2*^WT/WT^ (**c**) and *Katnal2*^Y86C/Y86C^ (**d**) spermatids, normal coupling of the centriole to the nuclear membrane was observed (black arrowheads). Docking of the centriole to the plasma membrane (white arrowhead) was frequently observed in *Katnal2*^WT/WT^ spermatids (**c**), but was never seen in *Katnal2*^Y86C/Y86C^ spermatids (**d**). Supernumerary centrioles (red arrowhead) were frequently observed in the cytoplasm of *Katnal2*^Y86C/Y86C^ (**d**) but not *Katnal2*^WT/WT^ (**c**) spermatids. Immunostaining of centrioles in isolated elongating spermatids of *Katnal2*^WT/WT^ (**e**) and *Katnal2*^Y86C/Y86C^ (**f**) mice confirmed abnormal duplication of centrioles (red arrowhead) in *Katnal2*^Y86C/Y86C^ mice. In (**e-f**) red represents centrioles as labelled by centrin immunostaining and blue represents DNA as labelled by DAPI. Scale bars in **c**–**f** = 2 μm. Primary antibody negative controls for (**a**–**b**) and (**e**–**f**) are included in S7 Fig.

Several severe abnormalities were, however, seen in all haploid germ cells. These included abnormal nuclear morphology (club-shaped, Fig 2G), the absence of sperm tail growth (see below) and spermiation failure (Fig 2I). Microtubule abnormalities were highlighted via the staining of testis sections for α-tubulin, as a marker of the manchette in particular (Fig 3B), and acetylated tubulin, as a marker of the axoneme at the core of the sperm tail (Fig 4A and 4B). Identical abnormalities were observed in *Katnal2*^*Y86C/KO*^ and *Katnal2*^*KO/KO*^ testes (S2 and S3 Figs).

In relation to sperm head shaping, the loss of KATNAL2 function resulted in abnormal manchette function and, ultimately, the constriction of elongated spermatid nuclei and abnormally long manchette microtubules (Fig 3A and 3B). The manchette is a transient microtubule-based structure involved in the shaping of the distal half of the sperm head and also acts as a platform for protein transport into the developing sperm tail [26,27]. A closer analysis of the step-by-step development of elongating spermatids at a light microscopic level indicated that while manchettes appeared to form at the appropriate time, the perinuclear ring failed to migrate distally, but maintained its ability to constrict in a development-dependent manner (Fig 3B). As a result, elongated spermatids developed a ‘knobby’ head morphology (Figs 2G versus 2F and 3A). In addition, during the later steps of elongated spermatid development, the microtubules of the manchette were abnormally long and took considerably longer to be disassembled (Fig 3A and 3B). Specifically, and as illustrated in Fig 3B, manchette length was normal in stage VIII (step 8) elongating spermatids, was obviously longer in stage X-XII tubules, and in contrast to the absence of manchettes in wild type stage I-II (step 14) tubules, *Katnal2*^*Y86C/Y86C*^ manchettes were clearly still present in mutant germ cells. These microtubule and head shaping abnormalities were confirmed in Staput isolated spermatids (S4 Fig). Notably, these abnormalities phenocopied those seen in *Katnb1* loss-of-function germs cells [24], suggesting that KATNAL2 and KATNB1 act in partnership within the manchette.

**Fig 5:**
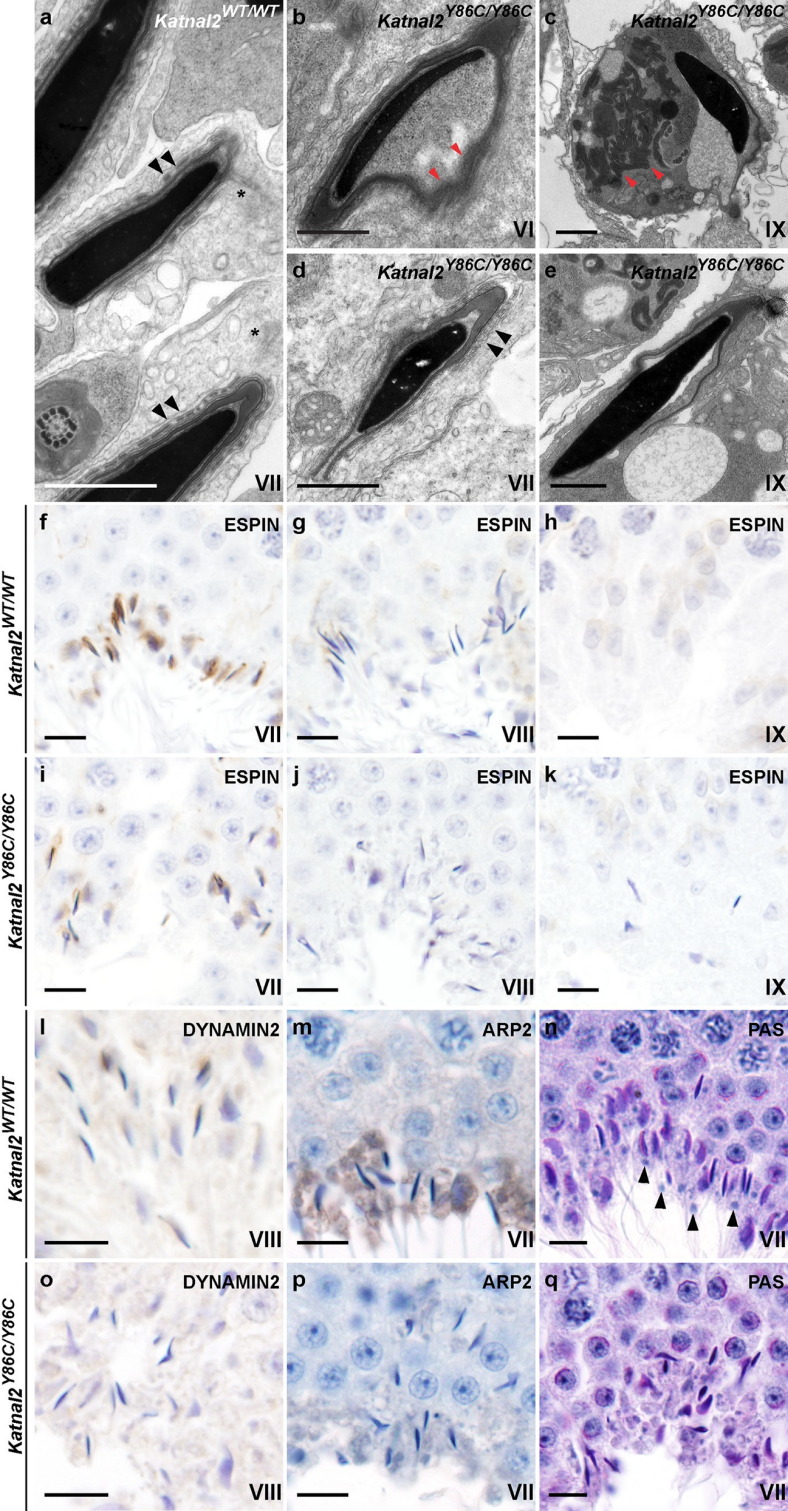
Tubulobulbar complex and residual body formation requires KATNAL2. (**a**–**e**) Electron microscopy showing elongated spermatid morphology during spermiation in *Katnal2*^*WT/WT*^ and *Katnal2*^*Y86C/Y86C*^ mice. Detachment of the acrosome (red arrowheads) from the nucleus was frequently observed in *Katnal2*^Y86C/Y86C^ (**b**) but not *Katnal2*^WT/WT^ (**a**) spermatids. Retention of cytoplasm was frequently seen in *Katnal2*^Y86C/Y86C^ spermatids that had failed spermiation (**c**). Within the cytoplasm of retained spermatids, disorganised outer dense fibres (red arrowheads) were frequently observed (**c**). Ectoplasmic specializations (black arrowheads) showed normal morphology in *Katnal2*^Y86C/Y86C^ (**d**) and *Katnal2*^WT/WT^ (**a**) spermatids, and were absent in *Katnal2*^Y86C/Y86C^ retained spermatids (**e**) indicating they were disassembled normally prior to spermiation. Tubulobulbar complexes (asterisks) were present in *Katnal2*^WT/WT^ (**a**) spermatids but were absent in *Katnal2*^Y86C/Y86C^ spermatids (**d**). Scale bars in **a**–**e** = 1 μm. Immunostaining of seminiferous tubules for the ectoplasmic specialization marker espin confirmed ectoplasmic specialisations were disassembled normally in *Katnal2*^WT/WT^ and *Katnal2*^Y86C/Y86C^ spermatids (**f**–**k**). Ectoplasmic specializations were present in early step 16 (stage VII) *Katnal2*^WT/WT^ (**f**) and *Katnal2*^Y86C/Y86C^ spermatids (**i**), but were absent from late step 16 (stage VIII) *Katnal2*^WT/WT^ (**g**) and *Katnal2*^Y86C/Y86C^ spermatids (**j**) and from retained (stage IX) *Katnal2*^Y86C/Y86C^ spermatids (**k**). Immunostaining of seminiferous tubules for the tubulobulbar complex markers dynamin-2 and ARP2 confirmed the absence of tubulobulbar complexes from *Katnal2*^Y86C/Y86C^ (**o**–**p**) but not *Katnal2*^WT/WT^ (**l**–**m**) spermatids. Residual body (arrowheads) formation in *Katnal2*^WT/WT^ (**n**) versus *Katnal2*^Y86C/Y86C^ (**q**) mice. Residual bodies were absent in *Katnal2*^Y86C/Y86C^ (**q**) mice. Scale bars in **f**–**q** = 10 μm. Primary antibody negative controls for (**f**–**m**) and (**o**–**p**) are included in S7 Fig.

In addition to the sperm head shaping abnormalities, *Katnal2*^*Y86C/Y86C*^ germ cells exhibited an almost complete absence of sperm tail growth i.e. >99% of cells (Fig 4A and 4B). At a sub-cellular level, as revealed by electron microscopy, the origin of this abnormality appeared to be a failure of centriole / basal body function. As shown in Fig 4C and 4D, in mutant germ cells the centriole, that should ultimately seed the axoneme of the sperm tail, migrated normally to the pole opposite the acrosome, matured normally into a basal body as indicated the presence of distal and sub-distal appendages, and docked normally with the nuclear membrane (Fig 4D). By contrast, the basal bodies failed to attach to the plasma membrane and there was a complete absence of microtubule extension, and thus axoneme development, in the overwhelming majority of cells (Fig 4D). Of interest, in many cases, elongating spermatids contained supernumerary centrioles (Fig 4D). Abnormal centriole duplication was confirmed with immunolabelling of isolated spermatids with the centriolar marker centrin (Fig 4E and 4F). Consistent with data from cell lines wherein it has been shown that time equivalent to approximately 1.5 cell divisions is required for centrioles to duplicate [28], very few duplicated centrioles were seen in round spermatids. Further, and consistent with data from Schatten and colleagues [29], who showed that centrioles in mouse elongated spermatids degrade, very few instances of centriole duplication were observed in elongated spermatids. Notably, the duplication of centrioles did not result in multiple basal bodies docking to the nuclear membrane indicating nuclear docking was not influenced by KATNAL2. These data do, however, indicate a role for KATNAL2 in suppressing centriole duplication in germ cells and is consistent with previously published data on KATNAL2 function in NIH3T3 cells and analogous to the supernumerary centrioles seen in *Katnb1* null neurons [22,30]. Despite the absence of axoneme extension, the formation of the outer dense fibers, that normally lie adjacent and parallel to the microtubules of the axoneme, was still initiated. These fibers were, however, massed within the cytoplasm of retained spermatids, rather than being within the flagellar compartment (Fig 5C).

Interestingly, the majority of elongated spermatids also displayed a detached acrosome (Fig 5B), indicating that KATNAL2, or KATNAL2-regulated structures, have a role in the deposition of ‘adhesive components’ into the acroplaxome. The acroplaxome is an electron dense structure that overlies the anterior pole of the sperm nucleus and is thought to play a pivotal role in acrosome formation and attachment [31,32]. This phenotype was not observed in *Katnb1* loss-of-function mice [24], suggesting acrosome attachment is KATNB1-independent.

Electron microscopy also revealed the origin of the spermiation defect in *Katnal2*^Y86C/Y86C^ mice as being in junction complexes between Sertoli cells and elongated spermatids. During spermiogenesis, spermatids are initially tethered to Sertoli cells by a modified adherens junction called the ectoplasmic specialization [33]. As spermiation proceeds, and spermatids are readied for their final disengagement and release from Sertoli cells, the ectoplasmic specializations are removed and replaced with a podosome-like endocytic structure called the tubulobulbar complex [34]. The tubulobulbar complex is proposed to be required for the removal of sperm-Sertoli cell adhesion structures and excess spermatid cytoplasm prior to sperm release. The removed excess cytoplasm, known as residual bodies, are phagocytosed by Sertoli cells after the sperm are released [33]. In wild type mouse germ cells, the tubulobulbar complexes form in stage VII and are present until sperm disengage from the seminiferous epithelium in late stage VIII. In *Katnal2*^*Y86C/Y86C*^ testis, the ectoplasmic specializations were formed and removed normally (Fig 5D and 5E), however, in all *Katnal2*^*Y86C/Y86C*^ spermatids observed by electron microscopy there was no evidence of tubulobulbar complex formation or the removal of excess germ cell cytoplasm i.e. no residual bodies formed and spermatids retained large amounts of cytoplasm (Fig 5C and 5Q). This interpretation was confirmed by immunolabelling of testes sections with espin, as a marker of the ectoplasmic specialization (Fig 5F–5K), and dynamin-2 and ARP2 as markers for the tubulobulbar complex (Fig 5L, 5M, 5O and 5P). Of note, in comparison to rats, which are the typical model used in tubulobulbar complex research, the tubulobulbar complexes of mice are considerably shorter (0.8–1.6 μm in mouse versus 3–5 μm in rat spermatids) and less numerous (6–10 in mouse versus up to 24 in rat spermatids) [35].

### Sperm formation and male fertility requires the co-ordinated action of both germ cell and Sertoli cell KATNAL2

**Fig 6:**
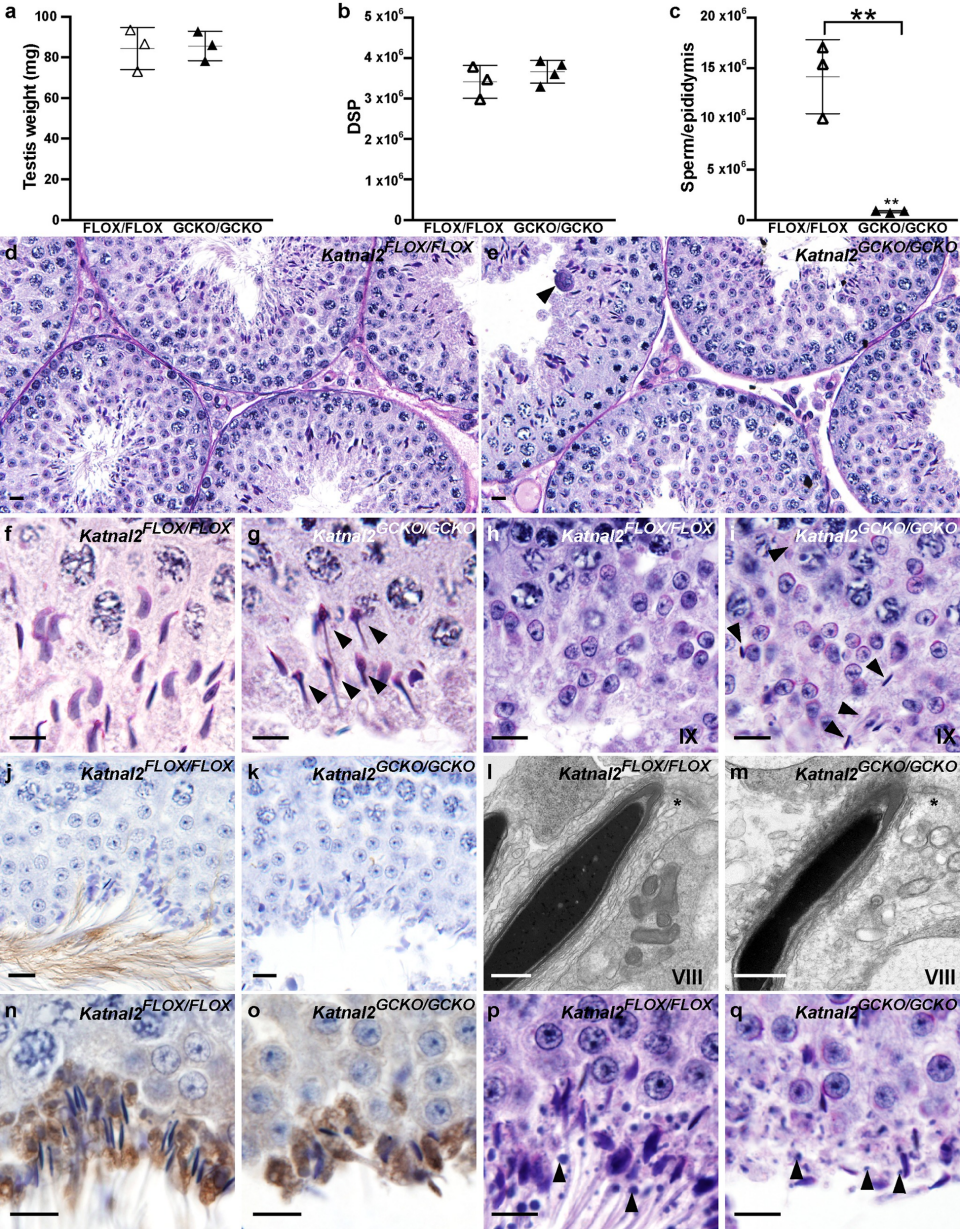
Spermatogenic defects in *Katnal2*^GCKO/GCKO^ mice. (**a**) Testis weight in *Katnal2*^FLOX/FLOX^ (white triangles) and *Katnal2*^GCKO/GCKO^ (black triangles) mice (n = 3/group). (**b**) Daily sperm output (DSP) in the testes of *Katnal2*^FLOX/FLOX^ (white triangles; n = 3) and *Katnal2*^GCKO/GCKO^ (black triangles; n = 4) mice. (**c**) Total epididymal sperm content of *Katnal2*^FLOX/FLOX^ (white triangles) and *Katnal2*^GCKO/GCKO^ (black triangles) mice (n = 3/group). Total epididymal sperm content was reduced by 94.1% in *Katnal2*^GCKO/GCKO^ mice compared to *Katnal2*^FLOX/FLOX^ mice. In **a**–**c** lines represent mean ± SD, ** p<0.01 compared to *Katnal2*^FLOX/FLOX^. Periodic acid Schiff’s (PAS) stained testis sections from *Katnal2*^FLOX/FLOX^ and *Katnal2*^GCKO/GCKOO^ mice (**d**–**i** and **p-q**). Low magnification view of seminiferous tubules in *Katnal2*^FLOX/FLOX^ (**d**) and *Katnal2*^GCKO/GCKO^ (**e**) mice. Multinucleated symplasts (arrowhead) were frequently observed in the *Katnal2*^GCKO/GCKO^ seminiferous epithelium. Elongating spermatids in *Katnal2*^FLOX/FLOX^ (**f**) versus *Katnal2*^GCKO/GCKO^ (**g**) mice. Abnormal nuclear morphology of spermatids (arrowheads) was frequently observed in *Katnal2*^GCKO/GCKO^ mice. Spermiation in *Katnal2*^FLOX/FLOX^ (**h**) versus *Katnal2*^GCKO/GCKO^ (**i**) mice. Retained elongated spermatids (arrowheads) were often observed in stage IX tubules of *Katnal2*^GCKO/GCKO^ mice but were rarely observed in *Katnal2*^FLOX/FLOX^ mice. Acetylated tubulin immunolabelling as a marker for sperm tail presence in *Katnal2*^FLOX/FLOX^ (**j**) and *Katnal2*^GCKO/GCKO^ (**k**) testis sections. Sperm tail content was markedly reduced in *Katnal2*^GCKO/GCKO^ (**k**) versus *Katnal2*^WT/WT^ (**j**) mice. Scale bars in **d**–**k** = 10 μm. Electron microscopy of stage VIII tubulobulbar complexes (asterisks) in *Katnal2*^FLOX/FLOX^ (**l**) versus *Katnal2*^GCKO/GCKO^ (**m**) mice. Tubulobulbar complex formation was normal in *Katnal2*^GCKO/GCKO^ mice. Scale bars in **l**–**m** = 2 μm. Immunostaining of seminiferous tubules for the tubulobulbar complex marker ARP2 in *Katnal2*^FLOX/FLOX^ (**n**) versus *Katnal2*^GCKO/GCKO^ (**o**) mice further confirmed the presence of tubulobulbar complexes from *Katnal2*^GCKO/GCKO^ spermatids. Residual body (arrowheads) formation in *Katnal2*^FLOX/FLOX^ (**p**) versus *Katnal2*^GCKO/GCKO^ (**q**) mice. Residual body formation appeared normal in *Katnal2*^GCKO/GCKO^ mice. Scale bars in **n**–**q** = 10 μm. Primary antibody negative controls for (**j**–**k**) and (**n**–**o**) are included in S7 Fig.

In order to ascertain if the origin of the spermatogenesis defects in *Katnal2* mutant mice were due to germ cell autonomous effects or reliant on interactions between germ cells and their supporting Sertoli cells, germ cell-specific (*Katnal2*^*GCKO*^) knockout mice were produced. *Katnal2* deletion from germ cells was achieved using a *Stra8*-cre. Gene deletion was confirmed using quantitative PCR on purified germ cells (S5 Fig). *Katnal2*^*GCKO/GCKO*^ males were sterile and presented with an identical manchette and sperm tail phenotype to that seen in *Katnal2*^*Y86C/Y86C*^ males (Fig 6). Sperm were not released from the seminiferous epithelium i.e. *Katnal2*^*GCKO/GCKO*^ epididymides only contained 5.9% of the sperm seen in wild type littermates (Fig 6C). However, in contrast to *Katnal2*^*Y86C/Y86C*^ males, tubulobulbar complex and residual body formation was comparable to that seen in wild type mice as evidenced at electron microscopic level (Fig 6L and 6M) and following immunolabelling with the tubulobulbar complex marker ARP2 (Fig 6N and 6O). Collectively, these data raise the possibility that tubulobulbar complex formation, and the removal of excess germ cell cytoplasm, is dictated largely by Sertoli cell-derived KATNAL2.

### KATNAL2 can interact with KATNB1

As noted above, the manchette phenotype observed in *Katnal2*^*Y86C/Y86C*^ germ cells phenocopied that seen in *Katnb1*^*Taily/Taily*^ males [24] supporting the hypothesis that KATNAL2 and KATNB1 act in concert within the manchette. To explore the possibility of a physical interaction between KATNAL2 and KATNB1, we co-transfected pEGFP-*Katnal2* and pmCherry-*Katnb1* into HEK293T cells and performed co-immunoprecipitation assays. These data show that KATNAL2 and KATNB1 can interact (Fig 3C). In order to define the sites of KATNAL2-KATNB1 localisation within isolated germ cells, we performed *in situ* proximity ligation assays. Cells were post-stained with DAPI, to visualize the nucleus, for α-tubulin, to visualize microtubules and with PNA, to visualise the acrosome (Fig 3D). The specificity of the assay was confirmed by staining *Katnal2*^*KO/KO*^ spermatids in parallel (Fig 3D). Consistent with the phenotype data, in elongating spermatids the KATNAL2-KATNB1 signal co-localised with manchette microtubules (Fig 3D), thus supporting a role for this complex in the movement and dissolution of the manchette in the terminal steps of spermiogenesis. In contrast, there was no evidence of KATNAL2-KATNB1 complex localization to the centriole / basal body or tubulobulbar complexes. Consistent with this observation, while the *Katnb1* mutant mice [24] did present with sperm flagellum defects, these defects were overtly different from those seen in the *Katnal2* mutant germ cells described here. Notably, microtubules did extend from the basal body in *Katnb1* mutant spermatids, however, the resultant axonemes lacked individual or multiple microtubules—typically the central pair within the 9+2 microtubule structure of the motile axoneme [24,36]. In *Katnal2* mutant germ cells, by contrast, there was a complete absence of axoneme development. These data strongly suggest that KATNAL2 is required for multiple aspects of male haploid germ cell development, and depending on the cellular context, KATNAL2 may act in either a KATNB1-dependent (manchette function) and KATNB1-independent manner (acrosome and axoneme development).

### KATNAL2 does not sever microtubules composed of α- and β-tubulin but does interact with δ- and ε-tubulin

Aspects of the *Katnal2*^*Y86C/Y86C*^ phenotype, including the failure of manchette movement and delayed dissolution, are consistent with deficiencies in microtubule severing. In order to test if KATNAL2 can sever microtubules, HEK293T cells were stably transfected with full length *Katnal2* cDNA and protein expression induced using a cumate-inducible promoter. We found no evidence that KATNAL2 over-expression affected cell proliferation or survival (S6 Fig). These data are consistent with a recently published study showing KATNAL2 overexpression did not affect α-tubulin bulk or microtubule lattice morphology [17]. We, therefore, deduce that KATNAL2 does not indiscriminately sever microtubules made of α and β-tubulin. These results do not, however, rule out the possibility that KATNAL2 severing function may be specific to particular microtubule modifications or be co-factor-dependent.

**Fig 7:**
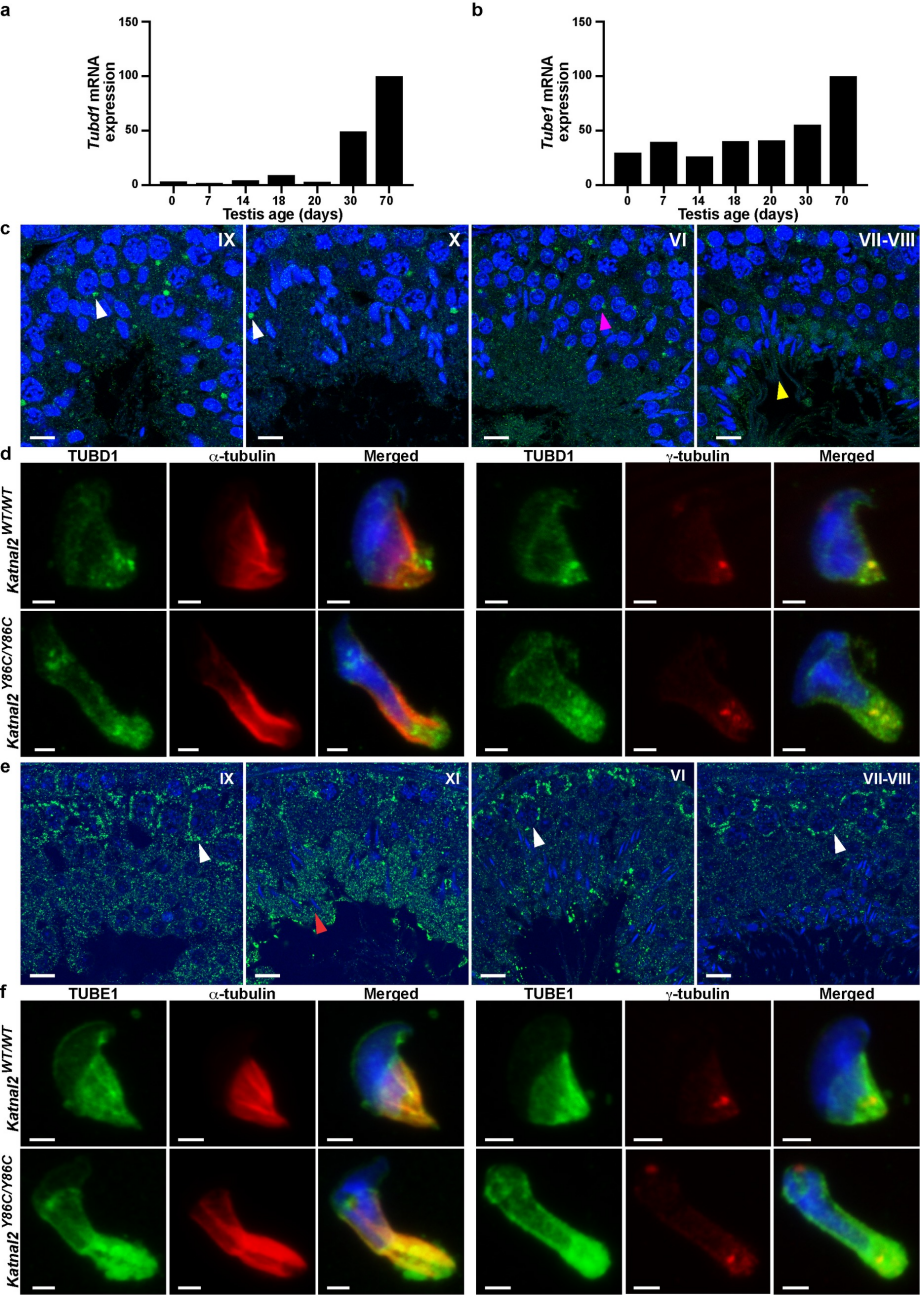
δ- and ε-tubulin are enriched in the adult testis. qPCR analysis of *Tubd1* (**a**) and *Tube1* (**b**) mRNA transcript levels in whole testis homogenates from *Katnal2*^WT/WT^ mice of various ages. Data are normalised to *Hprt* and presented relative to *Katnal2*^WT/WT^ day 70. δ-tubulin (TUBD1) (**c**) and ε-tubulin (TUBE1) (**e**) immunolabelling (green) in *Katnal2*^WT/WT^ testis sections. Examples of key cell types are indicated: pachytene spermatocytes (white arrowheads), round spermatids (magenta arrowhead), spermatid flagella (yellow arrowhead) and elongating spermatids (red arrowhead). Blue represents DNA as labelled by TOPRO. Scale bars in **c** and **e** = 10 μm. TUBD1 (**d**) and TUBE1 (**f**) localisation in *Katnal2*^WT/WT^ and *Katnal2*^Y86C/Y86C^ isolated elongating spermatids. Cells were counterstained with DAPI to visualise DNA, and either α-tubulin or γ-tubulin immunolabelling as markers for microtubules and centrioles, respectively. Scale bars in **d** and **f** = 2 μm. Primary antibody negative controls for (**c**–**f**) are included in S7 Fig.

The absence of severing of α-β tubulin polymers, but the presence of phenotypes consistent with microtubule severing, raise the hypothesis that KATNAL2 may mediate some of its functions by acting upon other tubulin subunits; specifically, δ-tubulin and ε-tubulin, which are enriched in male germ cells. As shown in Fig 7A, *Tubd1* (δ-tubulin) was up-regulated during the first wave of spermatogenesis at day 30, coinciding with the appearance of elongating spermatids, with a transient up-regulation at day 18 i.e. consistent with the appearance of late spermatocytes or early round spermatids. *Tube1* (ε-tubulin), however, showed a more consistent level of expression until it was up-regulated in day 30 testes, consistent with the appearance of elongating spermatids (Fig 7B). Immunolabelling of testis sections for δ-tubulin, showed it to be intensely localised to a granule within the cytoplasm of meiotic and haploid germ cells, and less intensely along the spermatid flagella (Fig 7C). Consistent with previously published research [37,38], and as most clearly observed within isolated spermatids, δ-tubulin localized to the perinuclear ring and microtubules of the manchette, in addition to the centrioles (Fig 7D, co-labelled with γ-tubulin). By contrast ε-tubulin had not previously been studied in the testis, but is known to localise to the centriole and its depletion blocks centriole duplication in *Xenopus laevis* egg extracts and *Tetrahymena thermophile* [39,40]. Additionally, ε-tubulin mutation and silencing in *Chlamydomonas reinhardtii and Paramecium tetraurelia*, results in malformed, dysfunctional centrioles [41,42]. Immunohistochemical labelling of testis sections for ε-tubulin revealed it to be present in the cytoplasm of all germ cell types (Fig 7E). ε-tubulin was particularly enriched in the cytoplasm of pachytene spermatocytes (stages I-X) and in the cytoplasm of spermatids as they underwent nuclear remodelling starting from stage X (step 10). Analysis of the subcellular localisation of ε-tubulin in isolated wild-type elongated spermatids, revealed ε-tubulin localised to the centriolar/basal body region and the manchette (Fig 7F).

**Fig 8:**
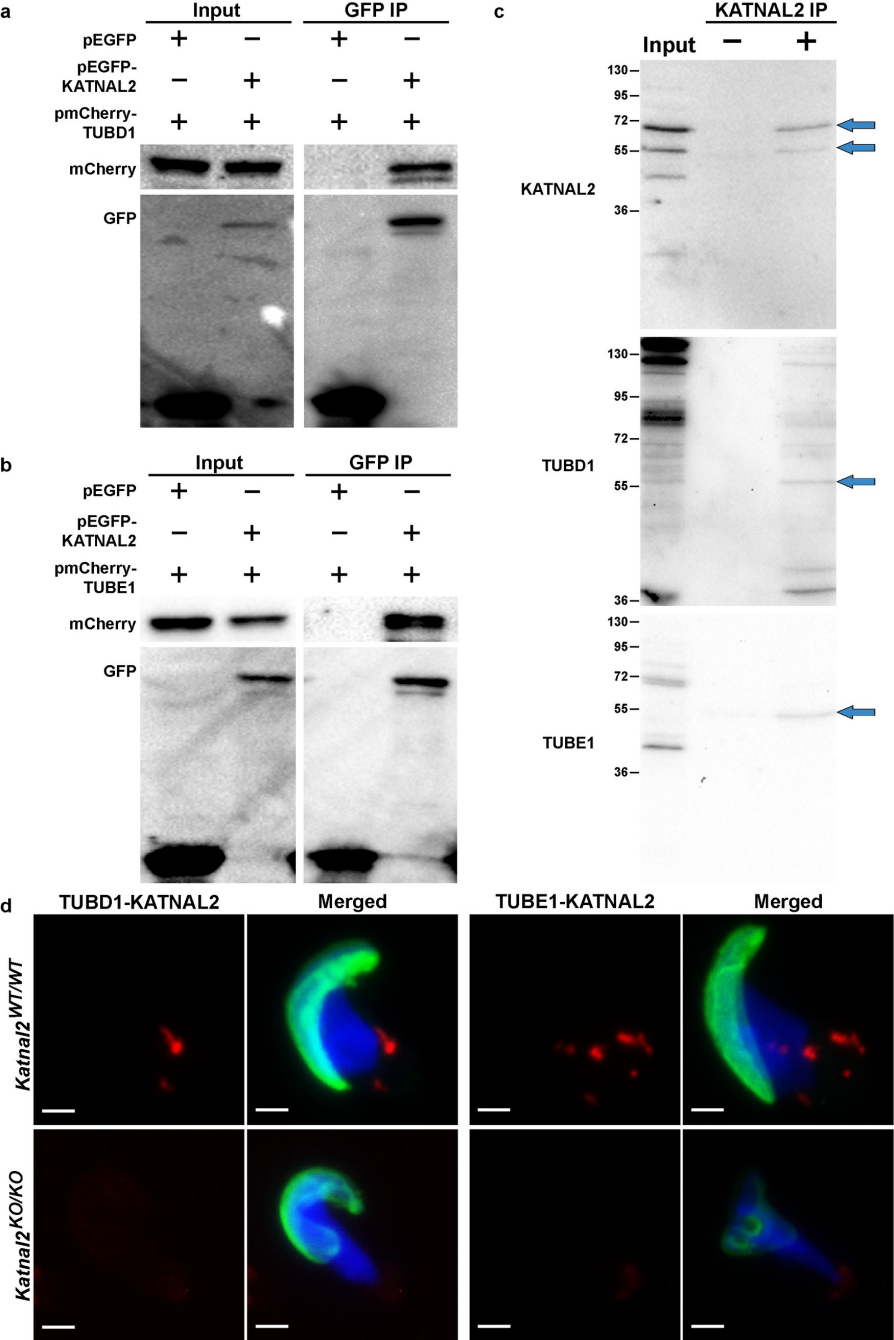
KATNAL2 interacts with δ- and ε-tubulin. KATNAL2 interaction with δ-tubulin (TUBD1) (**a**) and ε-tubulin (TUBE1) (**b**) was indicated by co-immunoprecipitation of pEGFP-KATNAL2-pmCherry-TUBD1 and pEGFP-KATNAL2-pmCherry-TUBE1 complexes respectively, using anti-GFP beads. (**c**) Interactions were confirmed by co-immunoprecipitation assays from mouse testis lysate and (**d**) *in situ* proximity ligation assays. (**a-b**) Input: whole cell lysate from transfected cells; GFP IP: immunoprecipitation with GFP conjugated beads. (**a**) The left upper panel shows mCherry-TUBD1 was successfully transfected into both cell populations and the left lower panel shows EGFP and EGFP-KATNAL2 were successfully transfected into the desired cell population. The right upper panel confirmed mCherry-TUBD1 can bind to EGFP-KATNAL2, but not to EGFP. Right lower panel confirmed EGFP and EGFP-KATNAL2 proteins were successfully precipitated with GFP beads. (**b**) The left upper panel shows mCherry-TUBE1 was successfully transfected into both cell populations and the left lower panel shows EGFP and EGFP-KATNAL2 were successfully transfected into the desired cell population. The right upper panel confirmed mCherry-TUBE1 can bind to EGFP-KATNAL2, but not to EGFP. Right lower panel confirmed EGFP and EGFP-KATNAL2 proteins were successfully precipitated with GFP beads. (**c**) Input: whole testis lysate from adult mice; KATNAL2 IP: immunoprecipitation (**+**) and negative control immunoprecipitation (**-**) using an antibody directed against KATNAL2. Blots were probed with antibodies directed against KATNAL2, TUBD1 and TUBE1. (**d**) Representative images of *in situ* proximity ligation assays using antibodies directed against KATNAL2 and TUBD1 and against KATNAL2 and TUBE1 in *Katnal2*^WT/WT^ isolated elongating spermatids. The specificity of labelling was assessed via the parallel labelling of *Katnal2*^KO/KO^ cells. Green represents the acrosome as labelled by PNA and blue represents DNA as labelled by DAPI. Manufacturer recommended negative controls are included in S8 Fig. Scale bars in **d** = 2 μm.

To test the hypothesis that KATNAL2 interacts with, and potentially regulates, δ- and ε-tubulin, we separately co-transfected pEGFP-*Katnal2* with pmCherry-*Tubd1* (δ-tubulin) and pmCherry-*Tube1* (ε-tubulin) into HEK293T cells and performed co-immunoprecipitation assays. As shown in Fig 8A and 8B KATNAL2 can bind to both δ- and ε-tubulin. This result was confirmed using co-immunoprecipitation of KATNAL2-TUBD1 and KATNAL2-TUBE1 complexes from testis tissue (Fig 8C). In accordance, *in situ* proximity ligation assays in spermatids revealed the presence of KATNAL2-δ-tubulin complexes at the base of the spermatid nucleus, and KATNAL2-ε-tubulin complexes in the manchette and pericentriolar region (Fig 8D). The specificity of these assays was confirmed by the parallel labelling of *Katnal2*^*KO/KO*^ spermatids (Fig 8D). Collectively, these data raise the novel possibility that KATNAL2 regulates δ- and ε-tubulin to facilitate manchette and basal body function in the male germ line. The mechanism underlying this regulation will be the subject of future studies.

## Discussion

Spermiogenesis is the dramatic morphogenesis of haploid round germ cells into the structurally replete sperm. This necessitates the formation of an acrosome, the condensation of the haploid genome into its species-specific head shape, and the development of a highly complex tail /flagellum made up of several thousand different proteins. Many of these processes are underpinned by microtubule-based structures, for both protein transport and structural support, and involve a series of complex and dynamic interactions between the germ cell and supporting Sertoli cell. Here we show that KATNAL2 is critical for numerous aspects of this developmental process, including the initiation of the axoneme from the basal body, suppression of the spermatid centriole duplication cycle, sperm nuclear sculpting via the manchette, the attachment of the acrosome to the nucleus and the tethering of elongated spermatids to Sertoli cells via the tubulobulbar complex and ultimately sperm release via spermiation. Our data suggest that, depending on context, these functions are achieved by KATNAL2 acting in partnership with KATNB1, a known regulator of other katanin severing proteins [6,9,17], or via KATNB1-independent mechanisms. Our data further suggest a role for KATNAL2 in regulating δ- and ε-tubulin over the classical α-β-tubulin polymers that make up most microtubules.

Loss of functional KATNAL2 results in several phenotypes that are outwardly consistent with a loss of microtubule regulation e.g. abnormal elongation and delayed disassembly of the manchette microtubules and a failure of the manchette to move distally during elongated spermatid development. Some, but not all of these phenotypes, phenocopy those seen in our *Katnb1* mutant mice, notably the manchette movement and dissolution [24], thus suggesting KATNB1 and KATNAL2 act in partnership to achieve this function. The exact mechanism by which the manchette forms and moves during spermiogenesis is unknown, however, some exceptional electron microscopy done by Russell and colleagues in rodents indicated that the perinuclear ring and the inner-most microtubules of the manchette are tethered to the nuclear membrane by short rod-like structures, of unknown composition, that progressively ‘unzip’ as the manchette moves distally [43]. It is proposed that the progressive constriction and distal movement of the perinuclear ring and microtubule skirt of the manchette, which occurs in parallel with the exchange of nuclear histones for protamines ‘sets’ the species-specific shape of the sperm nucleus. The absence of manchette movement, as shown in the *azh* mouse [44], and our *Katnal2* and *Katnb1* mutant mice, enforces an extended association between the microtubules of the manchette and the nucleus and a resultant ‘knobby’ and elongated nuclear phenotype (see Fig 2 and S4 Fig herein and [24]). Our data suggest that the ‘unzipping’ of the rod-like structures is achieved by the KATNAL2-KATNB1 complex. The precise mechanism by which this occurs remains to be determined.

Our data also indicate that other aspects of KATNAL2, notably its actions at the basal body, are achieved in a KATNB1-independent manner. KATNAL2-KATNB1 complexes were not localized to the basal body, despite clear regulation of the basal body by KATNAL2 in spermatids. This regulation is consistent with recent data suggesting that KATNAL2 interacts with the well-characterized centriole proteins CEP97, CEP295 and CDK5RAP2 [17].

There are three known families of microtubule severing enzymes, katanins, spastins and fidgetins, which together with VPS4 proteins, comprise the meiotic clade of the AAA+ superfamily [45]. AAA microtubule severing proteins, including KATNA1 and spastin, typically function by oligomerising into hexamers to form a central pore that is believed to attach to the acidic C-terminal tails of α- or β-tubulin subunits within the microtubule polymer [8,46]. Following ATP hydrolysis, and a resultant conformational change, the hexamer is believed to ‘tug’ on the tubulin tail thus destabilizing it from the associated polymer, leading to microtubule severing [46].

*Katnal2* is a paralogue of the microtubule severing enzymes *Katna1* and *Katnal1*, and as such, had been presumed to have microtubule-severing capacity. Despite several attempts, including herein, to test if KATNAL2 severs α-β tubulin polymers in a manner consist with its microtubule severing paralogues, such activity has not been demonstrated [17,22]. KATNAL2 is thus, not an indiscriminate microtubule severing protein. KATNAL2 does, however, possess a highly conserved AAA domain, compatible with microtubule-severing activity in other AAA microtubule severing proteins. Outside this domain, however, KATNAL2 shows marked divergence in sequence, raising the possibility of an alternate mechanism to achieve microtubule severing or a different function [5].

Towards the goal of revealing this function, our data shows that KATNAL2 can bind to the non-classical tubulin subunits δ- and ε-tubulin, and that such interactions occur in both the manchette and the basal body region of haploid male germ cells. In support of this being a functionally meaningful interaction, studies done in *Chlamydamonas* show that loss of ε-tubulin function results in the mislocalization of PF15 (the orthologue of KATNB1), and by extension KAT1, the ancestral homologue of all of the p60 severing subunits, including KATNAL2, from the basal body [47]. As shown here, and consistent with previous publications [37,38], δ-tubulin is a component of both the manchette perinuclear ring and microtubule ‘skirt’. Both δ- and ε-tubulin are localized to the basal body (Fig 7), and we provide the first evidence that ε-tubulin localises to the manchette. Thus, we propose that KATNAL2 acts within the manchette and basal body to modify δ- and/or ε-tubulin function and that the absence of such modifications underpin the *Katnal2* mutant germ cell phenotypes described here. In further support of this hypothesis, ε-tubulin has been shown to be a critical regulator of the centriole duplication cycle in *Xenopu*s [39] and *Tetrahymena* [40]. How this would be achieved remains to be determined. δ- and ε-tubulin are presumed not to form polymers independently, although structural analysis suggests they may bind to the minus and plus end of α-β microtubules respectively, and that they both may be involved in lateral interactions between microtubules [48].

Mouse KATNAL2 is produced in multiple isoforms (Fig 1), some of which lack the AAA domain and are thus likely to lack any form of severing activity. Such forms may act in a dominant-negative manner and are consistent with the perhaps obvious statement that any form of tubulin severing activity must be tightly regulated. Several of these isoforms are highly enriched within particular germ cell types, further suggesting that their activity may be beyond simply switching on and off tubulin modifications. Notably, all KATNAL2 isoforms lack an overt VPS4 domain and the Microtubule-Interacting and Trafficking (MIT) domain seen in other AAA microtubule-severing enzymes. As exemplified in recent research in *Drosophila*, the MIT domain enhances katanin p60 severing activity (and negatively regulates its abundance), however it is dispensable for severing [10]. Interestingly, several of the KATNAL2 isoforms contain a LisH motif, which is not observed in other microtubule severing type proteins (Fig 1). LisH motifs have been implicated in protein localization and the regulation of microtubule dynamics [49,50] and, as such may substitute for the lack of the MIT domain. Defining the function of individual subunits will necessitate the production of isoform-specific antibodies and the identification of cell lines containing the context specific binding partners.

KATNAL2 is also an essential component of the mysterious process of spermiation and is required for tubulobulbar complex formation. The tubulobulbar complex is a podosome-like projection, between the maturing germ cells and the supporting Sertoli cells in the final steps of development, that is proposed to be involved in the removal of excess cytoplasm, thus ensuring the stream lined nature of the final sperm [33]. Our data strongly suggests that this interpretation is valid. Other publications have also suggested that the tubulobulbar complex is necessary for the removal of an earlier germ-Sertoli adhesion complex, the ectoplasmic specialization (reviewed in [34]). Intriguingly, our data, suggests ectoplasmic specialization removal can occur normally in the absence of tubulobulbar complexes.

Collectively, our data establish KATNAL2 as a critical regulator of multiple aspects of haploid male germ cell development including sperm head shaping, acrosome attachment, tail growth and Sertoli-germ cell adhesion. These data, and germ cells in particular, provide an ideal model within which to elucidate key elements of the biochemistry and cell biology around KATNAL2 of relevance to all KATNAL2 containing cells including neurons and ciliated cells [18–21]. This study, and our previous research, also provides a dramatic example of the sub-specialisation of katanin function with increasing cellular complexity. While in plants, all functions are achieved by a single katanin severing protein, mammals, and notably spermatogenesis, requires the co-ordinated action of multiple katanin severing proteins during meiosis, in haploid germ cells and in the supporting Sertoli cells.

## Materials and methods

### Ethics statement

All animal procedures were approved by the Monash Animal Experimentation Ethics Committee and conducted in accordance with Australian NHMRC Guidelines on Ethics in Animal Experimentation.

### Mouse model production and phenotypic analysis

Mice containing a Y86C mutation in the *Katnal2* gene were obtained from the Australian Phenomics Network missense mutation library (https://databases.apf.edu.au/mutations/). These mice were produced using N-ethyl-nitrosourea (ENU) mutagenesis and then the resultant mutations identified using a massively parallel sequencing method as outlined previously [51]. Mice were maintained on a C57BL/6J background and potential passenger mutations eliminated via breeding onto wild type mice from a pure C57BL/6J colony. The effect of the Y86C mutation on *Katnal2* mRNA and protein stability was determined by qPCR and western blotting as described below.

The *Katnal2* knockout mouse line was generated at the Australian Phenomics Network (APN) Monash University Node using a EUCOMM knockout first conditional-ready ES clone (EPD0656_4_A03). To disrupt the *Katnal2* gene (ENSMUSG00000025420), the FRT-LacZ-loxP-Neo-FRT-loxP-*Katnal2*-*exon3*-loxP cassette was inserted in intron 2 of the *Katnal2* gene. The straight knockout allele resulted in truncated mRNA containing exons 1–2 (ENSMUSE00000700583 and ENSMUSE00001233754), which encoded the first 40 N-terminal amino acids of the protein. Mice were maintained on a C57BL/6N background. The veracity of the *Katnal2* null model was confirmed by qPCR, western blotting and immunohistochemistry as described below. To generate a germ cell-specific *Katnal2* knockout line, a three step breeding strategy was used: (i) *Katnal2*^*KO/WT*^ mice were crossed with a transgenic line carrying the Flp recombinase gene to produce the *Katnal2* Flox allele i.e. exon 3 (ENSMUSE00001268471) flanked by loxP sites, (ii) *Katnal2*^*Flox/Flox*^ females were mated with a *Stra8-Cre* male [52] to produce *Katnal2*^*WT/Del*,*Stra8-Cre+*^ males, (iii) *Katnal2*^*WT/Del*,*Stra8-Cre+*^ males were mated with *Katnal2*^*Flox/Flox*^ females to produce germ cell-specific knockout (*Katnal2*^*Del/Del*, *Stra8-Cre+*^) progeny. Excision of exon 3 in this manner resulted in a translational frameshift and a premature stop codon in exon 4 (ENSMUSE00000098115). Efficiency of excision was determined by qPCR of isolated cell populations as described below (S5 Fig).

Mouse genotypes were determined from tail biopsies using real time PCR with specific probes designed for each allele (Transnetyx, Cordova, TN). Primer and probe sequences were as listed in S1 Table.

### Quantitative qPCR

Total RNA was extracted using Trizol reagent (Invitrogen) and converted to cDNA using SuperScript III (Invitrogen). A PCR primer set was designed spanning part of exon 5 and exon 6 of the *Katnal2* gene to allow detection of the four *Katnal2* splice variants affected by the Y86C mutation and *Katnal2* gene trap cassette. To verify excision of exon 3 in the germ cell specific *Katnal2* knockout line, a PCR primer set was designed spanning part of exon 2 and exon 3 of *Katnal2*. All *Katnal2* qPCRs were performed using Brilliant Fast SYBR Green qPCR master mix (Stratagene), in the Agilent Mx3000P qPCR system. *Gapdh* and *Hprt* expression were used as references for gene expression analysis. SYBR Green qPCR primer sequences and cycling parameters were as listed in S2 Table. The relative expression of *Tubd1* and *Tube1* was defined using TaqMan Assays (Applied Biosystems) Mm00444851_m1 and Mm01179881_m1 respectively, and normalised against *Hprt* (Mm00446968_m1). TaqMan qPCRs were performed in the Agilent Mx3000P qPCR system: 1cycle, 95ºC, 10 min; 40 cycles, 95ºC, 15 sec, 60ºC, 1 min. Differential expression for all qPCRs was analysed using the 2ΔΔ^CT^ method [53].

### Infertility characterization

The male infertility phenotypes in the *Katnal2* mutant mouse lines were defined as outlined in [54]. Fertility testing was conducted in male and female mice of ≥ 7 and ≥ 8 weeks-of-age respectively, wherein mutant males were mated with wild type females, or *vice versa*, over a period of 3 months. Plugging was monitored as an indication of normal mating behaviour. Daily sperm production and total epididymal sperm content were determined using the Triton X-100 nuclear solubilisation method as described previously (n≥3/genotype) [55]. Tissue histology was assessed in Bouin’s fixed tissue. Tissues were processed into paraffin blocks using standard methods. Periodic acid-Schiff’s staining was used to examine overall testis histology (n≥5/genotype). Ultra-structure was analysed using electron microscopy as outlined previously [56,57] (n = 3/genotype) and caudal epididymal sperm morphology was examined by haematoxylin and eosin staining of air-dried sperm smears. Germ cell apoptosis was evaluated by immunostaining for cleaved Caspase 3 and 9. The number of Caspase-positive cells in 100 seminiferous tubules per mouse was counted and averaged (n = 3/genotype).

### Germ cell isolation

Germ cells were isolated using the Staput method [24,58]. Spermatocytes, round spermatids and elongating spermatids were all at least 90% pure. For immunofluorescence, cells were fixed with either 4% paraformaldehyde or methanol (-20°C) for 10 minutes.

### Antibody production and use

A KATNAL2-specific polyclonal antibody was produced by immunising goats with a synthetic peptide (YYFVKFQKYPKVVKKAPDP) encoding amino acids 74–96 of the mouse KATNAL2 (Antibodies Australia, Werribee, Australia). Specific immunoglobulins were purified using the immunizing peptide as described previously [59]. Specificity was confirmed by immunohistochemistry and western blotting on wild type versus *Katnal2*^*KO/KO*^ testis.

Other primary antibodies used included those against acetylated-tubulin (T6793, Sigma, 1 in 4,000), α-tubulin (T5168, Sigma, 1 in 5,000), centrin (04–1624, Millipore, 1 μg ml^-1^), ARP2 (ab49674, Abcam, 0.23 μg ml^-1^), cleaved-caspase 3 (9664, Cell Signaling, 0.5 μg ml^-1^), cleaved-caspase 9 (9509, Cell Signalling, 1 μg ml^-1^), δ-tubulin (HPA023980, Sigma Aldrich, 4 μg ml^-1^; ab214216, Abcam, 5 μg ml^-1^), dynamin 2 (ab3457, Abcam, 0.08 μg ml^-1^), espin (611656, BD Transduction Laboratories, 1.25 μg ml^-1^), ε-tubulin (ab98833, Abcam, 8 μg ml^-1^), GFP (11814460001, Roche, 0.2 μg ml^-1^), γ-tubulin (ab27074, Abcam, 1 μg ml^-1^), katanin p80 (HPA041165, Sigma Aldrich, 2.4 μg ml^-1^), KATNAL2 N-terminus (SC-84855, Santa Cruz Biotechnology [24]), mCherry (ab167453, Abcam, 0.5 μg ml^-1^). Secondary antibodies included Alexa Fluor 488 donkey anti-goat (A11055, Invitrogen), Alexa Fluor 555 donkey anti-goat (A21432, Invitrogen), Alexa Fluor 488 donkey anti-mouse (A21202, Invitrogen), Alexa Fluor 555 donkey anti-mouse (A31570, Invitrogen), Alexa Fluor 647 donkey anti-mouse (A31571, Invitrogen), Alexa Fluor 488 donkey anti-rabbit (A21206, Invitrogen), Alexa Fluor 647 donkey anti-rabbit (A31573, Invitrogen), a goat anti-rabbit immunoglobulin horseradish peroxidase conjugate (Dako) and a rabbit anti-mouse immunoglobulin horseradish peroxidase conjugate (Dako). The specificity of immunolabelling was determined by staining parallel samples in the absence of primary antibody (S7 Fig) or where available knockout mouse tissue.

### Immunochemistry

Testis immunohistochemistry was conducted as previously described [59] for a minimum of three times per antibody. For KATNAL2 immunolabelling, an alternative antigen retrieval method was performed by microwaving sections in 50mM glycine (pH 3.5) for 10 minutes. To define protein localisation in isolated germ cells, cells were permeabilized in 0.2% Triton X-100 (PBS) for one hour at room temperature. Non-specific binding was minimized by blocking with CAS-Block (Invitrogen) for 30 minutes. Primary antibodies were diluted in DAKO antibody diluent and incubated overnight at 4°C. Secondary antibodies were diluted 1 in 500 and incubated at room temperature for one hour. DNA was visualized using 1 µg ml^-1^ 4’,6-diamidino-2-phenylindole (DAPI, Invitrogen) or 4 µM TO-PRO-3 Iodide (TOPRO, Thermo Scientific). Acrosomes were visualized using 0.5 µg ml^-1^ lectin peanut agglutinin (PNA), Alexa Fluor 488 conjugate (L21409, Life Technologies).

Duolink *in situ* proximity ligation assays were carried as per the manufacturer’s instructions (OLINK Biosciences). Briefly, isolated germ cells were permeabilized as described for immunofluorescence, followed by blocking with Duolink blocking solution (OLINK) for 30 minutes at 37°C. Primary antibodies were diluted in Duolink antibody diluent (OLINK) and incubated overnight at 4°C. Appropriate secondary antibodies conjugated to synthetic oligonucleotides (goat PLA probe PLUS, mouse PLA probe MINUS, rabbit PLA probe MINUS) were applied for one hour at 37°C. A ligation reaction was performed using the Duolink ligation solution and ligase (30 minutes at 37°C), which results in binding of the two PLA probes if they are less than 40 nanometres from one another. Rolling circle amplification and hybridisation with fluorescently labelled nucleotides was achieved using the Duolink amplification solution and polymerase (100 minutes at 37°C). The specificity of the assay was determined by staining parallel samples in the absence of either both or one of the primary antibodies (S8 Fig). To counterstain samples for microtubules, DNA and acrosomes, immunofluorescent staining was then conducted as described above.

Immunofluorescent images were taken with an SP8 confocal microscope (Leica Microsystems) in the Monash University Microimaging Facility. Z-stacks of isolated cells were collected at 0.3 μm or 0.5 μm intervals and assembled into flattened images using ImageJ 1.47N. Images were adjusted uniformly across the image and between groups.

### Western blotting

Proteins were extracted from whole testis homogenates and isolated germ cell populations using RIPA buffer (50mM Tris-HCL; 1% NP-40; 0.1% SDS; 0.5% sodium deoxycholate; 0.9% NaCl; 5mM EDTA ph 7.4) plus protease inhibitor cocktail (Calbiochem). Extracted protein was separated on a 12% SDS-PAGE gel, transferred to PVDF membranes and probed using primary antibodies. Bound antibody was detected using donkey anti-goat IgG HRP and donkey anti-rabbit IgG HRP (Dako) secondary antibodies with enhanced chemiluminescence ECL Plus detection kit (Thermo Scientific) or Clarity Max ECL substrate (BioRad).

### Co-immunoprecipitation

Full length mouse *Katnal*2, *Katnb1*, *Tubd1 and Tube1* were amplified from mouse testis using the following primers: *Katnal2* GGCGTGCTACTCTTCTCTCT and CTGCTGACATCCATGACACG; *Katnb1* GTCCAAGCCTGACATTCCAT and GGAGTTGCCCTGAGCAGTAA*; Tubd1* GAAAGCTAAGGCGGGAGTTTGGG and AGCTTTTCCTCTTGGCTTAGGG; *Tube1* GAAAGCTAAGGCGGGAGTTTGGG and CACTTGTGTAACACCATGTTGGGTTCTC. *Katnal2* was then cloned into the pEGFP-C1 expression vector (Clontech), whereas *Katnb1*, *Tubd1 and Tube1* were cloned into pmCherry-C1 expression vectors (Clontech). pEGFP-*Katnal2* was co-transfected with either pmCherry-*Katnb1*, pmCherry-*Tubd1* or pmCherry-*Tube1* into HEK293T cells (ATCC-CRL-3216) using Lipofectamine 3000 Reagent (L3000008, Life Technologies). Empty pEGFP-C1 vector was used as a negative control. After extracting protein from the transfected cells, co-immunoprecipitation was carried out using anti-GFP-Trap-A beads as per the manufacturer’s instructions (gta-100, Chromotek). The presence of recombinant KATNAL2, KATNB1, TUBD1 and TUBE1 proteins within the immunoprecipitate were determined using antibodies against GFP and mCherry.

An antibody targeted against the N-terminus of KATNAL2 (SC-84855, Santa Cruz Biotechnology), which is a region highly divergent from paralogues KATNA1 and KATNAL1, was used to co-immunoprecipitate KATNAL2, TUBD1 and TUBE1 from adult mouse testis lysates using the Pierce Co-Immunoprecipitation Kit (26149, Thermo Scientific), as per the manufacturer’s instructions. The presence of KATNAL2, TUBD1 and TUBE1 proteins within the immunoprecipitate was determined using western blotting and antibodies directed against KATNAL2, TUBD1 and TUBE1.

### KATNAL2 transfection into HEK293 cells

Over-expression of the full length KATNAL2 protein (61 kDa) was performed using the PiggyBac Transposon System according to the manufacturer’s instruction (SBI System Biosciences). The full-length mouse *Katnal2* cDNA was sub-cloned into the NheI and XhoI restrictions sites of the PBQM812A-1 PiggyBac cumate inducible plasmid using primers mKatnal2-NheI-Fw: ACACTTGCTAGCATGGAGCTTTCTTACCAGAC and mKatnal2-NotI-Rev: AAACAAGCGGCCGCTTATACAGACTCAAACTTCT. The Katnal2/PBQM812A-1 PiggyBac plasmid was co-transfected with a transposase vector to enable stable integration of the *Katnal2* cDNA into the HEK293T cell line (ATCC-CRL-3216). KATNAL2 expression was induced by the addition of cumate into culture media at 0.24 mg/ml. To determine the success of KATNAL2 expression, cells were collected 72 hours after the addition of induction media for RNA extraction and qPCR.

### Cell proliferation assay

To evaluate the effect of KATNAL2 overexpression on the cell cycle, a MTS assay was performed using the CellTiter 96 AQueous One Solution Cell Proliferation Assay (Promega). For each time point to be measured, a 96-well plate was seeded with 5x10^3^ cells per well. After a forty-eight hour incubation period at 37°C, media was supplemented with 0.24 mg/ml cumic acid and 0.25mM mg ATP, and controls were supplemented with 0.25mM mg ATP only. To conduct the MTS assay, CellTiter 96 AQueous One Solution Reagent was added directly to each well and the plate incubated for one hour at 37°C. Absorbance was measured at 490 nm.

### Statistics and reproducibility

Statistical significance was determined using an unpaired student’s T-tests in Graphpad Prism 6.0 with significance defined as a P value <0.05. For each animal experiment, including immunostaining, 3–9 animals were assessed per genotype.

## Supporting information

S1 Table Primer sequences for mouse genotyping. (DOCX)

S2 Table Primer sequences for SYBR Green qPCR. (DOCX)

S1 Fig Validation of the KATNAL2 antibody. Immunochemistry of KATNAL2 in *Katnal2*^WT/WT^ and *Katnal2*^KO/KO^ testis sections confirmed the specificity of the KATNAL2 antibody. Green represents KATNAL2 and blue represents DNA as labeled by DAPI. Scale bars = 10 μm.
(TIF)

S2 Fig Assessment and validation of the *KATNAL2*^*Y86C*^ mutation. (**a**–**b**) KATNAL2 protein expression in whole testis homogenates of *Katnal2*^Y86C/Y86C^ mice. (**a**) Western blot analysis of KATNAL2 protein expression in whole adult testis homogenates from *Katnal2*^WT/WT^ and *Katnal2*^Y86C/Y86C^ mice. (**b**) Densitometry of the western blot analysis of the 46 kDa KATNAL2 isoform expression in whole testis homogenates from *Katnal2*^Y86C/Y86C^ mice (black triangles; n = 3) relative to *Katnal2*^WT/WT^ (white triangles; n = 3). Lines represent mean ± SD, **** P<0.0001. (**c–k**) Genotype-phenotype confirmation of *Katnal2*^Y86C/Y86C^ mice. (**c**) Testis weight in *Katnal2*^WT/WT^ (white triangles; n = 3) and *Katnal2*^Y86C/KO^ (black triangles; n = 4) mice. (**d**) Daily sperm output (DSP) in the testes of *Katnal2*^WT/WT^ (white triangles; n = 3) and *Katnal2*^Y86C/KO^ (black triangles, n = 5) mice. (**e**) Total epididymal sperm content of *Katnal2*^WT/WT^ (white triangles; n = 3) and *Katnal2*^Y86C/KO^ (black triangles, n = 7) mice. Total epididymal sperm content was reduced by 94.5% in *Katnal2*^Y86C/KO^ mice compared to *Katnal2*^WT/WT^ mice. Lines represent mean ± SD, **** p<0.0001 compared to *Katnal2*^WT/WT^. Periodic acid Schiff’s (PAS) stained testis sections from *Katnal2*^WT/WT^ and *Katnal2*^Y86C/KO^ mice (**f**–**k**). Low magnification view of seminiferous tubules in *Katnal2*^WT/WT^ (**f**) and *Katnal2*^Y86C/KO^ (**g**) mice. Multinucleated symplasts (arrowhead) were frequently observed in the *Katnal2*^*Y86C*/KO^ seminiferous epithelium. Elongating spermatids in *Katnal2*^WT/WT^ (**h**) versus *Katnal2*^Y86C/KO^ (**i**) mice. Abnormal nuclear (club shaped) morphology of spermatids (arrowheads) was frequently observed in *Katnal2*^Y86C/KO^ mice. Spermiation in *Katnal2*^WT/WT^ (**i**) versus *Katnal2*^Y86C/KO^ (**k**) mice. Retained elongated spermatids (arrowheads) were often observed in stage IX tubules of *Katnal2*^Y86C/KO^ but were rarely observed in *Katnal2*^WT/WT^ mice. Scale bars in **f**–**k** = 10 μm.
(TIF)

S3 Fig Spermatogenic defects in *Katnal2*^KO/KO^ mice. (**a**). The *Katnal2* knockout first, conditional-ready allele. The FRT-LacZ-loxP-Neo-FRT-loxP-Katnal2-exon3-loxP cassette was inserted into intron 2 of the *Katnal2* gene. (**b**) Testis weight in *Katnal2*^WT/WT^ (white triangles; n = 5) and *Katnal2*^KO/KO^ (black triangles; n = 6) mice. (**c**) Daily sperm output (DSP) in the testes of *Katnal2*^WT/WT^ (white triangles) and *Katnal2*^KO/KO^ (black triangles) mice (n = 4/group). (**d**) Total epididymal sperm content of *Katnal2*^WT/WT^ (white triangles) and *Katnal2*^KO/KO^ (black triangles) mice (n = 5/group). Total epididymal sperm content was reduced by 96.8% in *Katnal2*^KO/KO^ mice compared to *Katnal2*^WT/WT^ mice. Lines represent mean ± SD, **** p<0.0001 compared to *Katnal2*^WT/WT^. Periodic acid Schiff’s (PAS) stained testis sections from *Katnal2*^WT/WT^ and *Katnal2*^KO/KO^ mice (**e**–**j**). Low magnification view of seminiferous tubules in *Katnal2*^WT/WT^ (**e**) and *Katnal2*^KO/KO^ (**f**) mice. Multinucleated symplasts (arrowhead) were frequently observed in the *Katnal2*^KO/KO^ seminiferous epithelium. Elongating spermatids in *Katnal2*^WT/WT^ (**g**) versus *Katnal2*^KO/KO^ (**h**) mice. Abnormal nuclear morphology of spermatids (arrowheads) was frequently observed in *Katnal2*^KO/KO^ mice. Spermiation in *Katnal2*^WT/WT^ (**i**) versus *Katnal2*^KO/KO^ (**j**) mice. Retained elongated spermatids (arrowheads) were often observed in stage IX tubules of *Katnal2*^KO/KO^ but were rarely observed in *Katnal2*^WT/WT^ mice. Scale bars in **e**–**j** = 10 μm.
(TIF)

S4 Fig Manchette structure in *Katnal2*^Y86C/Y86C^ mice. α-tubulin immunolabelling (green) as a marker for manchettes in *Katnal2*^WT/WT^ and *Katnal2*^Y86C/Y86C^ isolated spermatids. Elongating spermatids are shown in progressive steps of manchette development and spermatid elongation from left to right. Cells were counterstained with DAPI (blue) to visualize DNA. Scale bars = 2 μm.
(TIF)

S5 Fig Efficiency of *Katnal2* exon 3 excision in *Katnal2* GCKO mice. qPCR analysis of *Katnal2* transcript levels in isolated round spermatids from *Katnal2*^GCKO/GCKO^ mice relative to *Katnal2*^FLOX/FLOX^ (n = 3/genotype). *Katnal2* transcript levels in isolated round spermatids were reduced by 99.9% in *Katnal2*^GCKO/GCKO^ mice compared to *Katnal2*^FLOX/FLOX^ mice. Lines represent mean ± SD, *** p<0.001 compared to *Katnal2*^FLOX/FLOX^.
(TIF)

S6 Fig KATNAL2 is not essential for cell cycle progression. (**a**) Germ cell apoptosis in *Katnal2*^Y86C/Y86C^ mice. The average number of germ cells per seminiferous tubules positive for either cleaved-caspase 3 or cleaved-caspase 9 showed no difference between *Katnal2*^WT/WT^ and *Katnal2*^Y86C/Y86C^ mice (n = 3/genotype). Lines represent mean ± SD. (**b**) KATNAL2 overexpression has no influence cell cycle progression. HEK293T cells stably transfected with a cumate inducible *Katnal2* plasmid showed no difference in relative cell density using an MTS assay when cultured either in the absence (white triangles) or presence (black triangles) of cumate induction media at 24 hours, 48 hours, 72 hours and 96 hours after addition of media (n = 3/genotype). Lines represent mean ± SD.
(TIF)

S7 Fig Validation of immunolabelling specificity. The specificity of immunolabelling as shown by the staining of parallel samples in the absence of primary antibody. α-tubulin (**a**) and acetylated tubulin (**b**) testis immunohistochemistry and corresponding primary antibody negative controls. (**c**) Centrin immunolabelling (red) on isolated germ cells and corresponding primary antibody negative control. Espin (**d**), dynamin-2 (**e**) and ARP2 (**f**) testis immunohistochemistry and corresponding primary antibody negative controls. TUBD1 (**g**) and TUBE1 (**h**) testis immunolabelling and corresponding primary antibody negative controls. TUBD1 (green) and α-tubulin (red) (**i**), TUBD1 (green) and γ-tubulin (red) (**j**), TUBE1 (green) and α-tubulin (red) (**k**), and TUBE1 (green) and γ-tubulin (red) (**l**) immunolabelling on isolated germ cells and corresponding primary antibody negative controls. In (**a**–**b**) and (**d**–**f**) nuclei are counterstained with haematoxylin. In (**c**) and (**i**–**l**) blue represents DNA as labeled by DAPI. In (**g**–**h**) blue represents DNA as labeled by TOPRO. In (**a**–**b**) and (**d**–**h**) scale bars = 10 μm and in (**c**) and (**i**–**l**) scale bars = 2 μm.
(TIF)

S8 Fig Validation of *in situ* proximity ligation assay specificity. The specificity of the *in situ* proximity ligation assays as shown by the staining of parallel samples in the absence of either both or one of the primary antibodies. *In situ* proximity ligation assays using antibodies directed against KATNB1 and KATNAL2 (**a**), TUBD1 and KATNAL2 (**b**), and TUBE1 and KATNAL2 (**c**) in isolated *Katnal2*^WT/WT^ spermatids. (**a**–**c**) Single antibody control 1: Assay was conducted with only KATNAL2 antibody. Single antibody control 2: Assay was conducted with only (**a**) KATNB1 antibody, (**b**) TUBD1 antibody and (**c**) TUBE1 antibody. (**a**–**c**) Negative control: Assay was conducted in the absence of all primary antibodies. (**a**) Cells were counterstained for α-tubulin (cyan) as a marker of microtubules. (**a**–**c**) Blue represents DNA as labeled by DAPI and green represents the acrosome as labeled by PNA. Scale bars = 2 μm.
(TIF)

## References

[1] McNally FJ, Vale RD (1993) Identification of katanin, an ATPase that severs and disassembles stable microtubules. Cell 75: 419–429. 8221885 10.1016/0092-8674(93)90377-3

[2] Mains P, Kemphues K, Sprunger S, Sulston I, Wood W (1990) Mutations affecting the meiotic and mitotic divisions of the early Caenorhabditis elegans embryo. Genetics 126: 593–605. 2249759 10.1093/genetics/126.3.593 PMC1204215

[3] Clark-Maguire S, Mains PE (1994) mei-1, a gene required for meiotic spindle formation in Caenorhabditis elegans, is a member of a family of ATPases. Genetics 136: 533–546. 8150281 10.1093/genetics/136.2.533 PMC1205806

[4] Sharp DJ, Ross JL (2012) Microtubule-severing enzymes at the cutting edge. Journal of Cell Science 125: 2561–2569. doi: 10.1242/jcs.101139 22595526 10.1242/jcs.101139 PMC3403230

[5] Roll-Mecak A, McNally FJ (2010) Microtubule-severing enzymes. Current Opinion in Cell Biology 22: 96–103. doi: 10.1016/j.ceb.2009.11.001 19963362 10.1016/j.ceb.2009.11.001 PMC2822099

[6] Hartman JJ, Mahr J, McNally K, Okawa K, Iwamatsu A, et al (1998) Katanin, a microtubule-severing protein, is a novel AAA ATPase that targets to the centrosome using a WD40-containing subunit. Cell 93: 277–287. 9568719 10.1016/s0092-8674(00)81578-0

[7] Johjima A, Noi K, Nishikori S, Ogi H, Esaki M, et al (2015) Microtubule severing by katanin p60 AAA+ ATPase requires the C-terminal acidic tails of both α-and β-tubulins and basic amino acid residues in the AAA+ ring pore. Journal of Biological Chemistry 290: 11762–11770. doi: 10.1074/jbc.M114.614768 25805498 10.1074/jbc.M114.614768 PMC4416876

[8] Hartman JJ, Vale RD (1999) Microtubule disassembly by ATP-dependent oligomerization of the AAA enzyme katanin. Science 286: 782–785. 10531065 10.1126/science.286.5440.782

[9] McNally KP, Bazirgan OA, McNally FJ (2000) Two domains of p80 katanin regulate microtubule severing and spindle pole targeting by p60 katanin. Journal of Cell Science 113: 1623–1633. 10751153 10.1242/jcs.113.9.1623

[10] Grode KD, Rogers SL (2015) The Non-Catalytic Domains of Drosophila Katanin Regulate Its Abundance and Microtubule-Disassembly Activity. PLoS ONE 10: e0123912 doi: 10.1371/journal.pone.0123912 25886649 10.1371/journal.pone.0123912 PMC4401518

[11] McNally K, Audhya A, Oegema K, McNally FJ (2006) Katanin controls mitotic and meiotic spindle length. Journal of Cell Biology 175: 881–891. doi: 10.1083/jcb.200608117 17178907 10.1083/jcb.200608117 PMC2064698

[12] Srayko M, O'Toole ET, Hyman AA, Müller-Reichert T (2006) Katanin disrupts the microtubule lattice and increases polymer number in C. elegans meiosis. Current Biology 16: 1944–1949. doi: 10.1016/j.cub.2006.08.029 17027492 10.1016/j.cub.2006.08.029

[13] Nakamura M, Ehrhardt DW, Hashimoto T (2010) Microtubule and katanin-dependent dynamics of microtubule nucleation complexes in the acentrosomal Arabidopsis cortical array. Nature Cell Biology 12: 1064–1070. doi: 10.1038/ncb2110 20935636 10.1038/ncb2110

[14] Ahmad FJ, Yu W, McNally FJ, Baas PW (1999) An essential role for katanin in severing microtubules in the neuron. Journal of Cell Biology 145: 305–315. 10209026 10.1083/jcb.145.2.305 PMC2133110

[15] Stewart A, Tsubouchi A, Rolls MM, Tracey WD, Sherwood NT (2012) Katanin p60-like1 promotes microtubule growth and terminal dendrite stability in the larval class IV sensory neurons of Drosophila. Journal of Neuroscience 32: 11631–11642. doi: 10.1523/JNEUROSCI.0729-12.2012 22915107 10.1523/JNEUROSCI.0729-12.2012 PMC3495988

[16] Lee H-H, Jan LY, Jan Y-N (2009) Drosophila IKK-related kinase Ik2 and Katanin p60-like 1 regulate dendrite pruning of sensory neuron during metamorphosis. Proceedings of the National Academy of Sciences, USA 106: 6363–6368. 10.1073/pnas.0902051106 PMC2661847 19329489

[17] Cheung K, Senese S, Kuang J, Bui N, Ongpipattanakul C, et al (2016) Proteomic Analysis of the Mammalian Katanin Family of Microtubule-severing Enzymes Defines KATNBL1 as a Regulator of Mammalian Katanin Microtubule-severing. Molecular and Cellular Proteomics 15: 1658–1669. doi: 10.1074/mcp.M115.056465 26929214 10.1074/mcp.M115.056465 PMC4858946

[18] Neale BM, Kou Y, Liu L, Ma’Ayan A, Samocha KE, et al (2012) Patterns and rates of exonic de novo mutations in autism spectrum disorders. Nature 485: 242–245. doi: 10.1038/nature11011 22495311 10.1038/nature11011 PMC3613847

[19] De Rubeis S, He X, Goldberg AP, Poultney CS, Samocha K, et al (2014) Synaptic, transcriptional and chromatin genes disrupted in autism. Nature 515: 209–215. doi: 10.1038/nature13772 25363760 10.1038/nature13772 PMC4402723

[20] Sanders SJ, Murtha MT, Gupta AR, Murdoch JD, Raubeson MJ, et al (2012) De novo mutations revealed by whole-exome sequencing are strongly associated with autism. Nature 485: 237–241. doi: 10.1038/nature10945 22495306 10.1038/nature10945 PMC3667984

[21] Williams MR, Fricano-Kugler CJ, Getz SA, Skelton PD, Lee J, et al (2016) A Retroviral CRISPR-Cas9 System for Cellular Autism-Associated Phenotype Discovery in Developing Neurons. Scientific Reports 6: 25611 doi: 10.1038/srep25611 27161796 10.1038/srep25611 PMC4861960

[22] Ververis A, Christodoulou A, Christoforou M, Kamilari C, Lederer CW, et al (2016) A novel family of katanin-like 2 protein isoforms (KATNAL2), interacting with nucleotide-binding proteins Nubp1 and Nubp2, are key regulators of different MT-based processes in mammalian cells. Cellular and Molecular Life Sciences 73: 163–184. doi: 10.1007/s00018-015-1980-5 26153462 10.1007/s00018-015-1980-5 PMC11108477

[23] O’Donnell L O’Bryan MK (2014) Microtubules and spermatogenesis. Seminars in Cell and Developmental Biology 30: 45–54. doi: 10.1016/j.semcdb.2014.01.003 24440897 10.1016/j.semcdb.2014.01.003

[24] O'Donnell L, Rhodes D, Smith SJ, Merriner DJ, Clark BJ, et al (2012) An essential role for katanin p80 and microtubule severing in male gamete production. PLoS Genetics 8: e1002698 doi: 10.1371/journal.pgen.1002698 22654669 10.1371/journal.pgen.1002698 PMC3359970

[25] Smith LB, Milne L, Nelson N, Eddie S, Brown P, et al (2012) KATNAL1 regulation of sertoli cell microtubule dynamics is essential for spermiogenesis and male fertility. PLoS Genetics 8: e1002697 doi: 10.1371/journal.pgen.1002697 22654668 10.1371/journal.pgen.1002697 PMC3359976

[26] Lehti MS, Sironen A (2016) Formation and function of manchette and flagellum during spermatogenesis. Reproduction 106: 1683–1690. 10.1530/REP-15-0310 26792866

[27] Kierszenbaum AL, Rivkin E, Tres LL (2007) Molecular biology of sperm head shaping. Society of Reproduction and Fertility Supplement 65: 33 17644953

[28] Brito DA, Gouveia SM, Bettencourt-Dias M (2012) Deconstructing the centriole: structure and number control. Current Opinion in Cell Biology 24: 4–13. doi: 10.1016/j.ceb.2012.01.003 22321829 10.1016/j.ceb.2012.01.003

[29] Manandhar G, Sutovsky P, Joshi H, Stearns T, Schatten G (1998) Centrosome reduction during mouse spermiogenesis. Developmental Biology 203: 424–434. doi: 10.1006/dbio.1998.8947 9808791 10.1006/dbio.1998.8947

[30] Hu WF, Pomp O, Ben-Omran T, Kodani A, Henke K, et al (2014) Katanin p80 regulates human cortical development by limiting centriole and cilia number. Neuron 84: 1240–1257. doi: 10.1016/j.neuron.2014.12.017 25521379 10.1016/j.neuron.2014.12.017 PMC4485387

[31] Kierszenbaum AL, Tres LL (2004) The acrosome-acroplaxome-manchette complex and the shaping of the spermatid head. Archives of Histology and Cytology 67: 271–284. 15700535 10.1679/aohc.67.271

[32] Liu Y, DeBoer K, de Kretser DM, O’Donnell L, O’Connor AE, et al (2015) LRGUK-1 is required for basal body and manchette function during spermatogenesis and male fertility. PLoS Genetics 11: e1005090 doi: 10.1371/journal.pgen.1005090 25781171 10.1371/journal.pgen.1005090 PMC4363142

[33] O'Donnell L, Nicholls PK, O’Bryan MK, McLachlan RI, Stanton PG (2011) Spermiation: the process of sperm release. Spermatogenesis 1: 14–35. doi: 10.4161/spmg.1.1.14525 21866274 10.4161/spmg.1.1.14525 PMC3158646

[34] Vogl AW, Du M, Wang XY, J’Nelle SY (2014) Novel clathrin/actin-based endocytic machinery associated with junction turnover in the seminiferous epithelium. Seminars in Cell and Developmental Biology 30: 55–64. doi: 10.1016/j.semcdb.2013.11.002 24280271 10.1016/j.semcdb.2013.11.002

[35] Russell LD, Malone JP (1980) A study of Sertoli-spermatid tubulobulbar complexes in selected mammals. Tissue and Cell 12: 263–285. 6998046 10.1016/0040-8166(80)90005-1

[36] Dymek EE, Lefebvre PA, Smith EF (2004) PF15p is the Chlamydomonas homologue of the Katanin p80 subunit and is required for assembly of flagellar central microtubules. Eukaryotic Cell 3: 870–879. doi: 10.1128/EC.3.4.870-879.2004 15302820 10.1128/EC.3.4.870-879.2004 PMC500881

[37] Kato A, Nagata Y, Todokoro K (2004) δ-Tubulin is a component of intercellular bridges and both the early and mature perinuclear rings during spermatogenesis. Developmental Biology 269: 196–205. doi: 10.1016/j.ydbio.2004.01.026 15081367 10.1016/j.ydbio.2004.01.026

[38] Smrzka OW, Delgehyr N, Bornens M (2000) Tissue-specific expression and subcellular localisation of mammalian δ-tubulin. Current Biology 10: 413–416. 10753753 10.1016/s0960-9822(00)00418-8

[39] Chang P, Giddings TH, Winey M, Stearns T (2003) ε-Tubulin is required for centriole duplication and microtubule organization. Nature Cell Biology 5: 71–76. doi: 10.1038/ncb900 12510196 10.1038/ncb900

[40] Ross I, Clarissa C, Giddings TH, Winey M (2013) ε-tubulin is essential in Tetrahymena thermophila for the assembly and stability of basal bodies. Journal of Cell Science 126: 3441–3451. doi: 10.1242/jcs.128694 23704354 10.1242/jcs.128694 PMC3730247

[41] Dutcher SK, Morrissette NS, Preble AM, Rackley C, Stanga J (2002) ε-Tubulin is an essential component of the centriole. Molecular Biology of the Cell 13: 3859–3869. doi: 10.1091/mbc.E02-04-0205 12429830 10.1091/mbc.E02-04-0205 PMC133598

[42] Dupuis-Williams P, Fleury-Aubusson A, de Loubresse NG, Geoffroy H, Vayssié L, et al (2002) Functional role of ε-tubulin in the assembly of the centriolar microtubule scaffold. Journal of Cell Biology 158: 1183–1193. doi: 10.1083/jcb.200205028 12356863 10.1083/jcb.200205028 PMC2173240

[43] Russell LD, Russell JA, MacGregor GR, Meistrich ML (1991) Linkage of manchette microtubules to the nuclear envelope and observations of the role of the manchette in nuclear shaping during spermiogenesis in rodents. American Journal of Anatomy 192: 97–120. doi: 10.1002/aja.1001920202 1759685 10.1002/aja.1001920202

[44] Meistrich M, Trostle-Weige P, Russell L (1990) Abnormal manchette development in spermatids of azh/azh mutant mice. American Journal of Anatomy 188: 74–86. doi: 10.1002/aja.1001880109 2346121 10.1002/aja.1001880109

[45] Frickey T, Lupas AN (2004) Phylogenetic analysis of AAA proteins. Journal of Structural Biology 146: 2–10. doi: 10.1016/j.jsb.2003.11.020 15037233 10.1016/j.jsb.2003.11.020

[46] Roll-Mecak A, Vale RD (2008) Structural basis of microtubule severing by the hereditary spastic paraplegia protein spastin. Nature 451: 363–367. doi: 10.1038/nature06482 18202664 10.1038/nature06482 PMC2882799

[47] Esparza JM, O’Toole E, Li L, Giddings TH Jr, Kozak B, et al (2013) Katanin localization requires triplet microtubules in Chlamydomonas reinhardtii. PloS ONE 8: e53940 doi: 10.1371/journal.pone.0053940 23320108 10.1371/journal.pone.0053940 PMC3540033

[48] Inclán YF, Nogales E (2001) Structural models for the self-assembly and microtubule interactions of gamma-, delta-and epsilon-tubulin. Journal of Cell Science 114: 413–422. 11148142 10.1242/jcs.114.2.413

[49] Emes RD, Ponting CP (2001) A new sequence motif linking lissencephaly, Treacher Collins and oral–facial–digital type 1 syndromes, microtubule dynamics and cell migration. Human Molecular Genetics 10: 2813–2820. 11734546 10.1093/hmg/10.24.2813

[50] Gerlitz G, Darhin E, Giorgio G, Franco B, Reiner O (2005) Novel functional features of the Lis-H domain: role in protein dimerization, half-life and cellular localization. Cell Cycle 4: 1632–1640. doi: 10.4161/cc.4.11.2151 16258276 10.4161/cc.4.11.2151

[51] Andrews TD, Whittle B, Field M, Balakishnan B, Zhang Y, et al (2012) Massively parallel sequencing of the mouse exome to accurately identify rare, induced mutations: an immediate source for thousands of new mouse models. Open Biology 2: 120061 doi: 10.1098/rsob.120061 22724066 10.1098/rsob.120061 PMC3376740

[52] Sadate‐Ngatchou PI, Payne CJ, Dearth AT, Braun RE (2008) Cre recombinase activity specific to postnatal, premeiotic male germ cells in transgenic mice. Genesis 46: 738–742. doi: 10.1002/dvg.20437 18850594 10.1002/dvg.20437 PMC2837914

[53] Livak KJ, Schmittgen TD (2001) Analysis of Relative Gene Expression Data Using Real-Time Quantitative PCR and the 2− ΔΔCT Method. Methods 25: 402–408. doi: 10.1006/meth.2001.1262 11846609 10.1006/meth.2001.1262

[54] Borg CL, Wolski KM, Gibbs GM, O'Bryan MK (2009) Phenotyping male infertility in the mouse: how to get the most out of a ‘non-performer’. Human Reproduction Update 16: 205–224. doi: 10.1093/humupd/dmp032 19758979 10.1093/humupd/dmp032 PMC2816191

[55] Cotton L, Gibbs GM, Sanchez-Partida LG, Morrison JR, de Kretser DM, et al (2006) FGFR-1 signaling is involved in spermiogenesis and sperm capacitation. Journal of Cell Science 119: 75–84. doi: 10.1242/jcs.02704 16352663 10.1242/jcs.02704

[56] Pesch S, Bergmann M (2006) Structure of mammalian spermatozoa in respect to viability, fertility and cryopreservation. Micron 37: 597–612. doi: 10.1016/j.micron.2006.02.006 16621580 10.1016/j.micron.2006.02.006

[57] Arsov T, Silva DG, O’Bryan MK, Sainsbury A, Lee NJ, et al (2006) Fat aussie—a new Alstrom syndrome mouse showing a critical role for ALMS1 in obesity, diabetes, and spermatogenesis. Molecular Endocrinology 20: 1610–1622. doi: 10.1210/me.2005-0494 16513793 10.1210/me.2005-0494

[58] Romrell L, Bellvé AR, Fawcett DW (1976) Separation of mouse spermatogenic cells by sedimentation velocity: a morphological characterization. Developmental Biology 49: 119–131. 176074 10.1016/0012-1606(76)90262-1

[59] Jamsai D, Bianco DM, Smith SJ, Merriner DJ, Ly-Huynh JD, et al (2008) Characterization of gametogenetin 1 (GGN1) and its potential role in male fertility through the interaction with the ion channel regulator, cysteine-rich secretory protein 2 (CRISP2) in the sperm tail. Reproduction 135: 751–759. doi: 10.1530/REP-07-0485 18502891 10.1530/REP-07-0485

