# Supplementary material for: Katanin-like 2 (KATNAL2) functions in multiple aspects of haploid male germ cell development in the mouse
Source: PLoS Genet. 2017 Nov 14;13(11):e1007078. doi: 10.1371/journal.pgen.1007078 (PMC5705150; doi:10.1371/journal.pgen.1007078)
Supplement: S1 Table — (DOCX) [file pgen.1007078.s001.docx]

**S1 Table: Primer sequences for mouse genotyping**

| **Allele** | **Assay name** | **Primers and probes** |
| --- | --- | --- |
| *Katnal2^Y86C/WT^* | Katnal2-1 MUT | Forward Primer: GACCTGGAAACTATTTTGATGGAATATGAGA  Reverse: AGCATCATTTCTAGCACAAACCTATGT  Reporter-1: AAGTTCCAGAAGTACCCCAAA  Reporter-2: CCAGAAGTGCCCCAAA |
| *Katnal2^KO/WT^* | Katnal2-2 WT | Forward Primer: GAACAGTGGGAACATCACCAGAT  Reverse Primer: CAACGCTGTCAACTGAAACACT  Reporter: TCCGTCTGGAAGCTAC |
|  | LAC Z | Forward Primer: CGATCGTAATCACCCGAGTGT  Reverse Primer: CCGTGGCCTGACTCATTCC  Reporter: CCAGCGACCAGATGAT |
| *Katnal2^Flox/WT^* | Katnal2-2 WT | Forward Primer: GAACAGTGGGAACATCACCAGAT  Reverse Primer: CAACGCTGTCAACTGAAACACT  Reporter: TCCGTCTGGAAGCTAC |
|  | L1L2-Bact-P MD | Forward Primer: GCTGGCGCCGGAAC  Reverse Primer: GCGACTATAGAGATATCAACCACTTTGT  Reporter: AAGCTGGGTCTAGATATC |
|  | LAC Z | Forward Primer: CGATCGTAATCACCCGAGTGT  Reverse Primer: CCGTGGCCTGACTCATTCC  Reporter: CCAGCGACCAGATGAT |
| *Katnal2^Flox/Flox^* | Katnal2-2 WT | Forward Primer: GAACAGTGGGAACATCACCAGAT  Reverse Primer: CAACGCTGTCAACTGAAACACT  Reporter: TCCGTCTGGAAGCTAC |
|  | L1L2-Bact-P MD | Forward Primer: GCTGGCGCCGGAAC  Reverse Primer: GCGACTATAGAGATATCAACCACTTTGT  Reporter: AAGCTGGGTCTAGATATC |
| *Katnal2^Flox/Del, Stra8-Cre+^* | Katnal2-2 WT | Forward Primer: GAACAGTGGGAACATCACCAGAT  Reverse Primer: CAACGCTGTCAACTGAAACACT  Reporter: TCCGTCTGGAAGCTAC |
|  | L1L2-Bact-P MD | Forward Primer: GCTGGCGCCGGAAC  Reverse Primer: GCGACTATAGAGATATCAACCACTTTGT  Reporter: AAGCTGGGTCTAGATATC |
|  | iCre | Forward Primer: TCCTGGGCATTGCCTACAAC  Reverse Primer: CTTCACTCTGATTCTGGCAATTTCG  Reporter: ACCCTGCTGCGCATTG |
