# Supplementary material for: Katanin-like 2 (KATNAL2) functions in multiple aspects of haploid male germ cell development in the mouse
Source: PLoS Genet. 2017 Nov 14;13(11):e1007078. doi: 10.1371/journal.pgen.1007078 (PMC5705150; doi:10.1371/journal.pgen.1007078)
Supplement: S2 Table — (DOCX) [file pgen.1007078.s002.docx]

**S2 Table: Primer sequences for SYBR Green qPCR**

| **Primer Set** | **Primer Sequences** | **Cycling Parameters** |
| --- | --- | --- |
| ***Katnal2* Exon 2-3** | Forward Primer: AAGGCTTTGGAGGAGGAGAC  Reverse Primer: GGGGCCTTCTTAACCACTTT | 50°C, 2min, 1 cycle; 95°C, 2 min, 1 cycle ; 95°C, 15 sec; 60°C, 1 min, repeat 40 cycles |
| ***Katnal2* Exon 5-6** | Forward Primer: CAGGGGACACCAAATCTGTC  Reverse Primer: CTGGCTGGTCTTTGTGGATT | 95°C, 2 min, 1 cycle; 95°C, 05 sec; 59°C, 30 sec; 72°C, 30 sec, repeat 45 cycles; 95°C, 15 sec; 60°C, 45 sec; 95°C, 05 sec, 1 cycle. |
