# Supplementary figures and images for: Katanin-like 2 (KATNAL2) functions in multiple aspects of haploid male germ cell development in the mouse
Source: PLoS Genet. 2017 Nov 14;13(11):e1007078. doi: 10.1371/journal.pgen.1007078 (PMC5705150; doi:10.1371/journal.pgen.1007078)

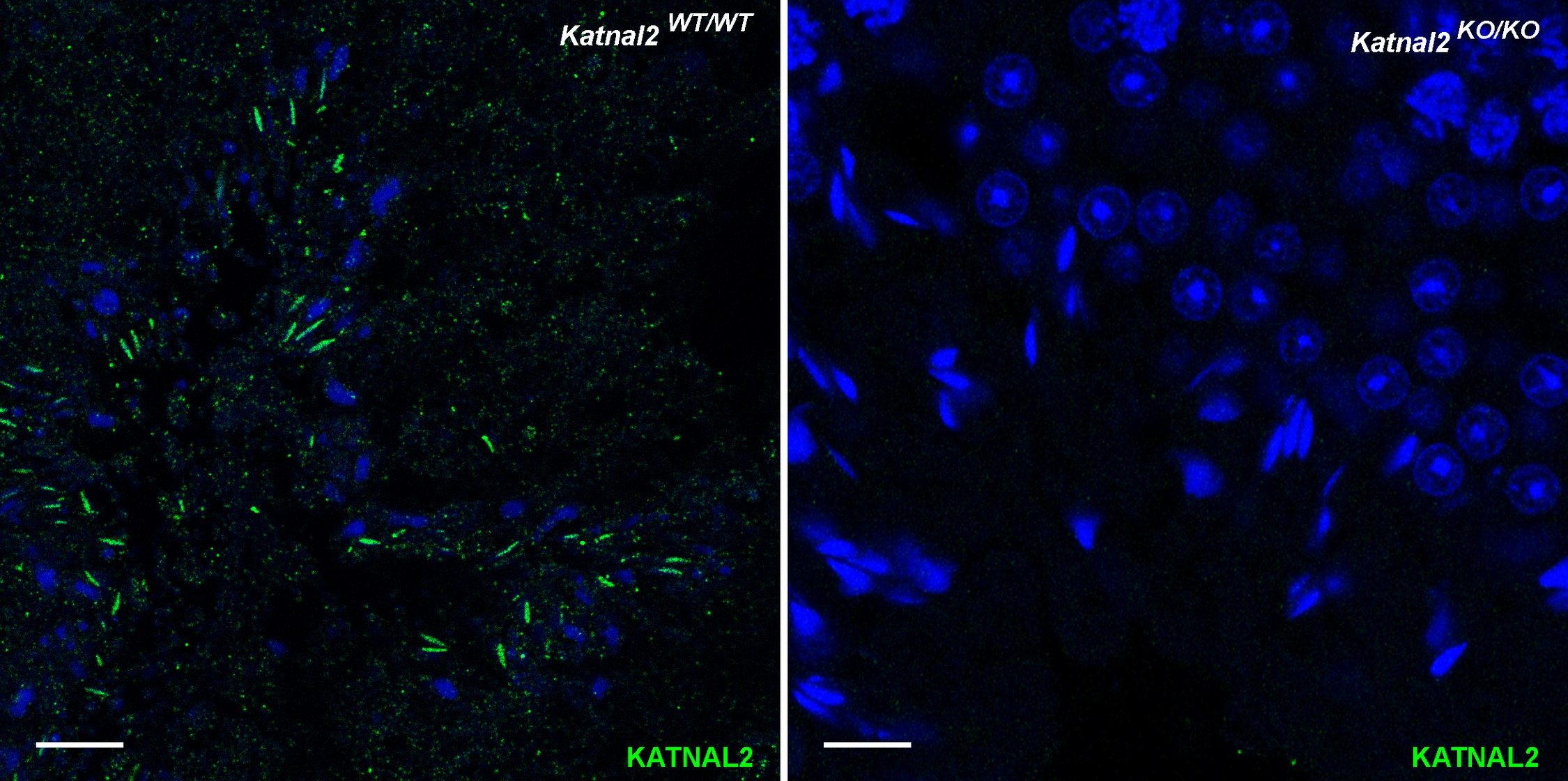

Supplement: S1 Fig — Immunochemistry of KATNAL2 in Katnal2WT/WT and Katnal2KO/KO testis sections confirmed the specificity of the KATNAL2 antibody. Green represents KATNAL2 and blue represents DNA as labeled by DAPI. Scale bars = 10 μm. (TIF) [file pgen.1007078.s003.tif]

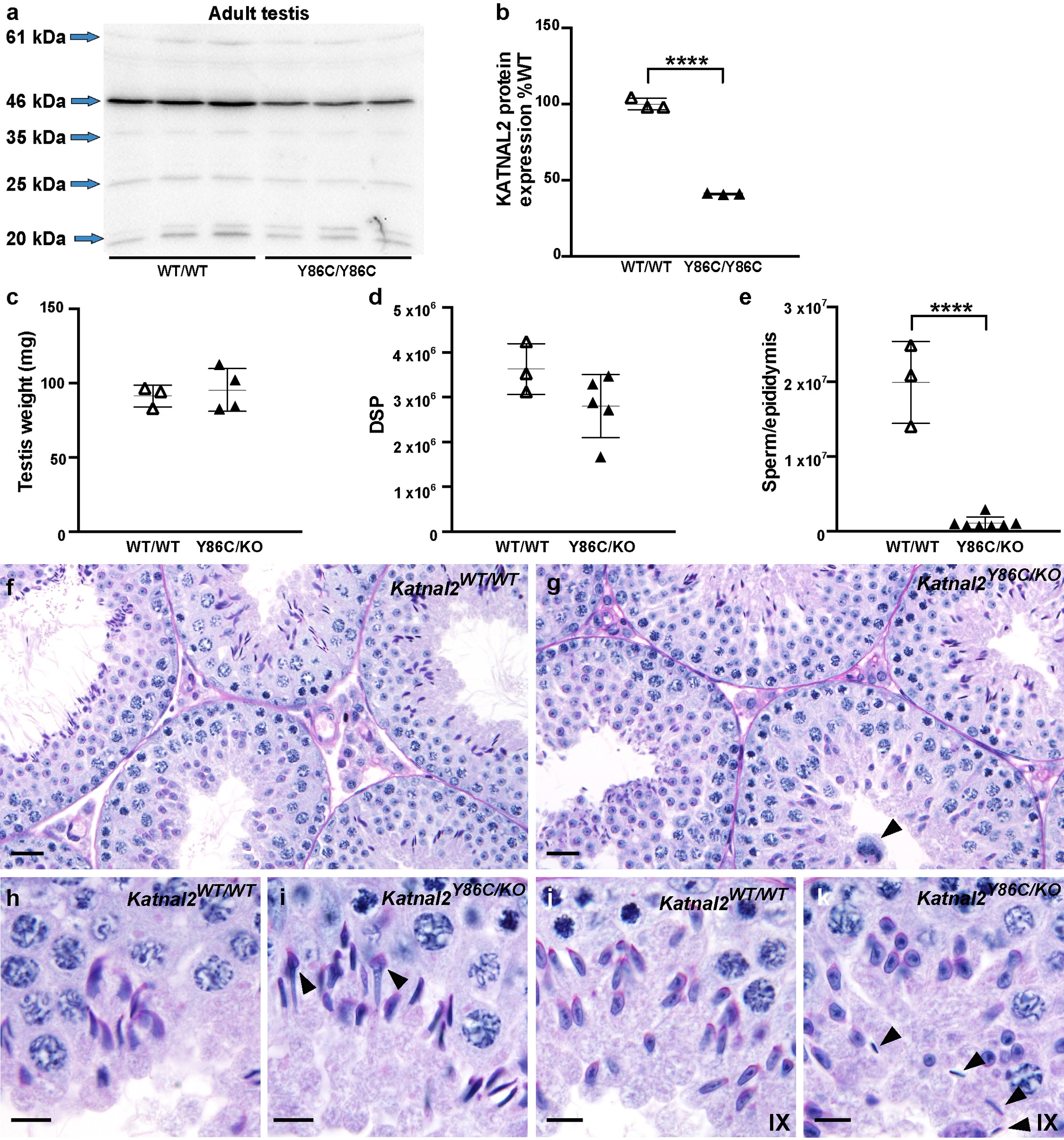

Supplement: S2 Fig — (a–b) KATNAL2 protein expression in whole testis homogenates of Katnal2Y86C/Y86C mice. (a) Western blot analysis of KATNAL2 protein expression in whole adult testis homogenates from Katnal2WT/WT and Katnal2Y86C/Y86C mice. (b) Densitometry of the western blot analysis of the 46 kDa KATNAL2 isoform expression in whole testis homogenates from Katnal2Y86C/Y86C mice (black triangles; n = 3) relative to Katnal2WT/WT (white triangles; n = 3). Lines represent mean ± SD, **** P<0.0001. (c–k) Genotype-phenotype confirmation of Katnal2Y86C/Y86C mice. (c) Testis weight in Katnal2WT/WT (white triangles; n = 3) and Katnal2Y86C/KO (black triangles; n = 4) mice. (d) Daily sperm output (DSP) in the testes of Katnal2WT/WT (white triangles; n = 3) and Katnal2Y86C/KO (black triangles, n = 5) mice. (e) Total epididymal sperm content of Katnal2WT/WT (white triangles; n = 3) and Katnal2Y86C/KO (black triangles, n = 7) mice. Total epididymal sperm content was reduced by 94.5% in Katnal2Y86C/KO mice compared to Katnal2WT/WT mice. Lines represent mean ± SD, **** p<0.0001 compared to Katnal2WT/WT. Periodic acid Schiff’s (PAS) stained testis sections from Katnal2WT/WT and Katnal2Y86C/KO mice (f–k). Low magnification view of seminiferous tubules in Katnal2WT/WT (f) and Katnal2Y86C/KO (g) mice. Multinucleated symplasts (arrowhead) were frequently observed in the Katnal2Y86C/KO seminiferous epithelium. Elongating spermatids in Katnal2WT/WT (h) versus Katnal2Y86C/KO (i) mice. Abnormal nuclear (club shaped) morphology of spermatids (arrowheads) was frequently observed in Katnal2Y86C/KO mice. Spermiation in Katnal2WT/WT (i) versus Katnal2Y86C/KO (k) mice. Retained elongated spermatids (arrowheads) were often observed in stage IX tubules of Katnal2Y86C/KO but were rarely observed in Katnal2WT/WT mice. Scale bars in f–k = 10 μm. (TIF) [file pgen.1007078.s004.tif]

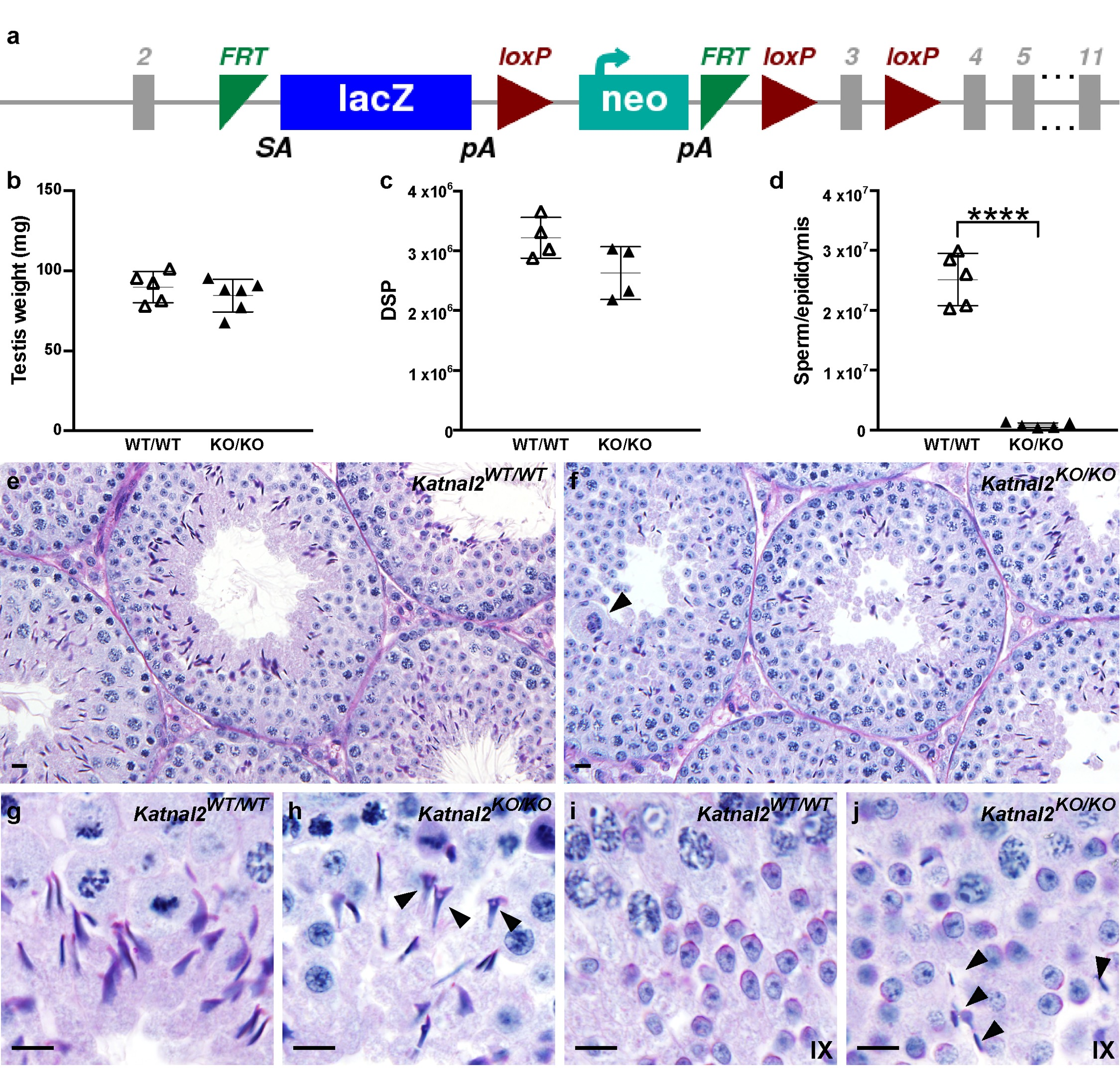

Supplement: S3 Fig — (a). The Katnal2 knockout first, conditional-ready allele. The FRT-LacZ-loxP-Neo-FRT-loxP-Katnal2-exon3-loxP cassette was inserted into intron 2 of the Katnal2 gene. (b) Testis weight in Katnal2WT/WT (white triangles; n = 5) and Katnal2KO/KO (black triangles; n = 6) mice. (c) Daily sperm output (DSP) in the testes of Katnal2WT/WT (white triangles) and Katnal2KO/KO (black triangles) mice (n = 4/group). (d) Total epididymal sperm content of Katnal2WT/WT (white triangles) and Katnal2KO/KO (black triangles) mice (n = 5/group). Total epididymal sperm content was reduced by 96.8% in Katnal2KO/KO mice compared to Katnal2WT/WT mice. Lines represent mean ± SD, **** p<0.0001 compared to Katnal2WT/WT. Periodic acid Schiff’s (PAS) stained testis sections from Katnal2WT/WT and Katnal2KO/KO mice (e–j). Low magnification view of seminiferous tubules in Katnal2WT/WT (e) and Katnal2KO/KO (f) mice. Multinucleated symplasts (arrowhead) were frequently observed in the Katnal2KO/KO seminiferous epithelium. Elongating spermatids in Katnal2WT/WT (g) versus Katnal2KO/KO (h) mice. Abnormal nuclear morphology of spermatids (arrowheads) was frequently observed in Katnal2KO/KO mice. Spermiation in Katnal2WT/WT (i) versus Katnal2KO/KO (j) mice. Retained elongated spermatids (arrowheads) were often observed in stage IX tubules of Katnal2KO/KO but were rarely observed in Katnal2WT/WT mice. Scale bars in e–j = 10 μm. (TIF) [file pgen.1007078.s005.tif]

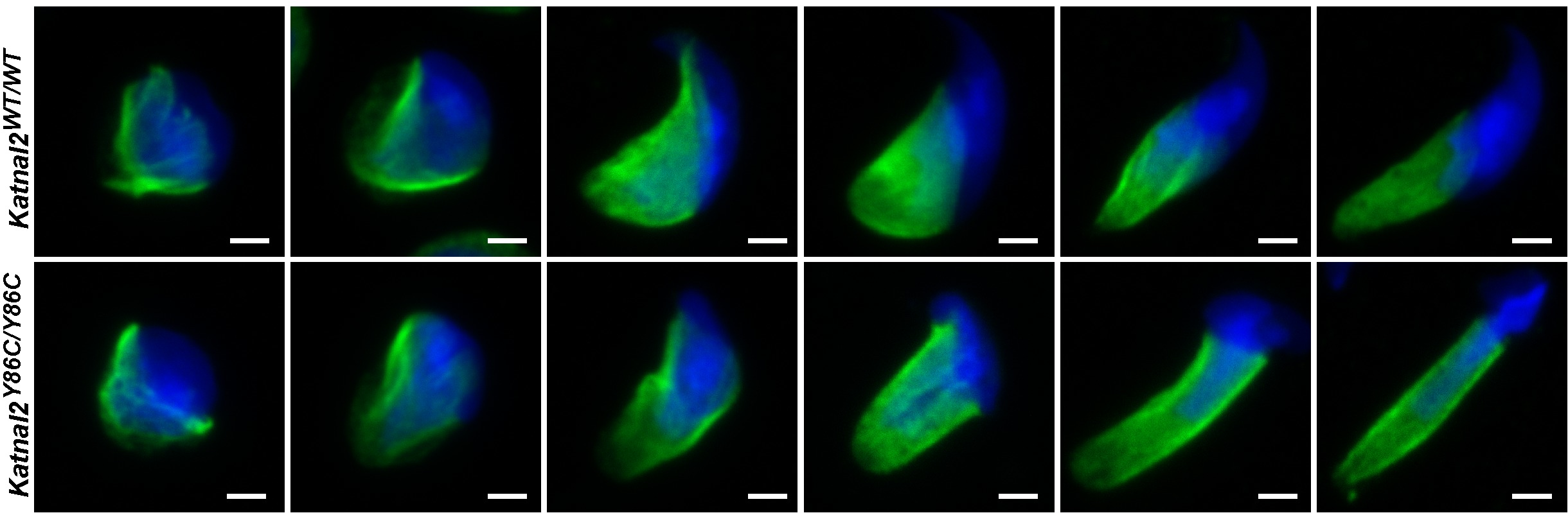

Supplement: S4 Fig — α-tubulin immunolabelling (green) as a marker for manchettes in Katnal2WT/WT and Katnal2Y86C/Y86C isolated spermatids. Elongating spermatids are shown in progressive steps of manchette development and spermatid elongation from left to right. Cells were counterstained with DAPI (blue) to visualize DNA. Scale bars = 2 μm. (TIF) [file pgen.1007078.s006.tif]

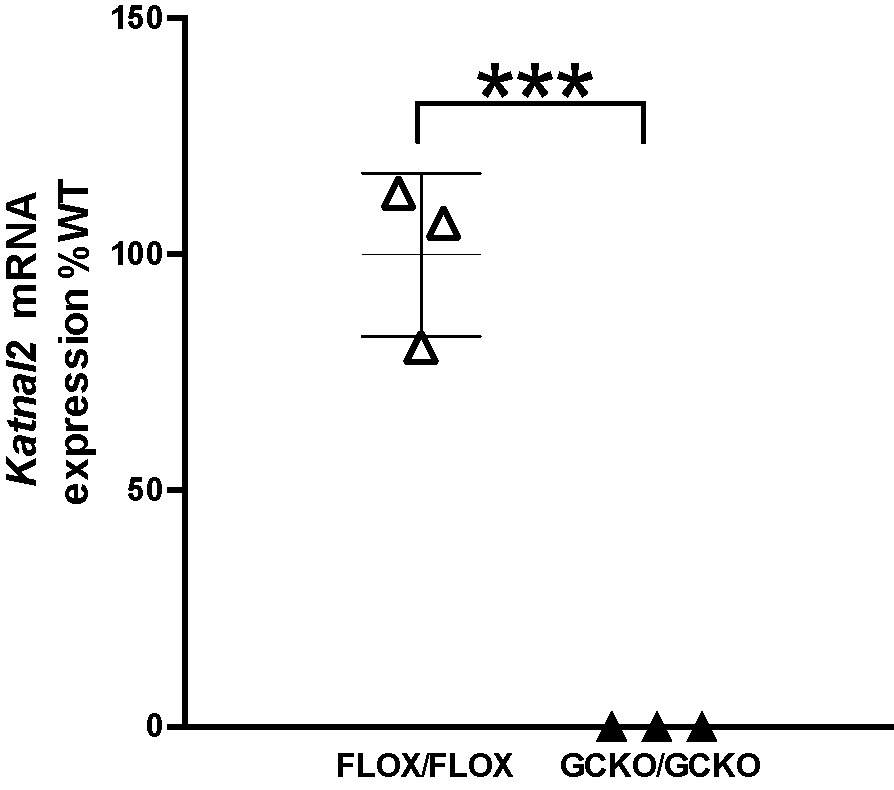

Supplement: S5 Fig — qPCR analysis of Katnal2 transcript levels in isolated round spermatids from Katnal2GCKO/GCKO mice relative to Katnal2FLOX/FLOX (n = 3/genotype). Katnal2 transcript levels in isolated round spermatids were reduced by 99.9% in Katnal2GCKO/GCKO mice compared to Katnal2FLOX/FLOX mice. Lines represent mean ± SD, *** p<0.001 compared to Katnal2FLOX/FLOX. (TIF) [file pgen.1007078.s007.tif]

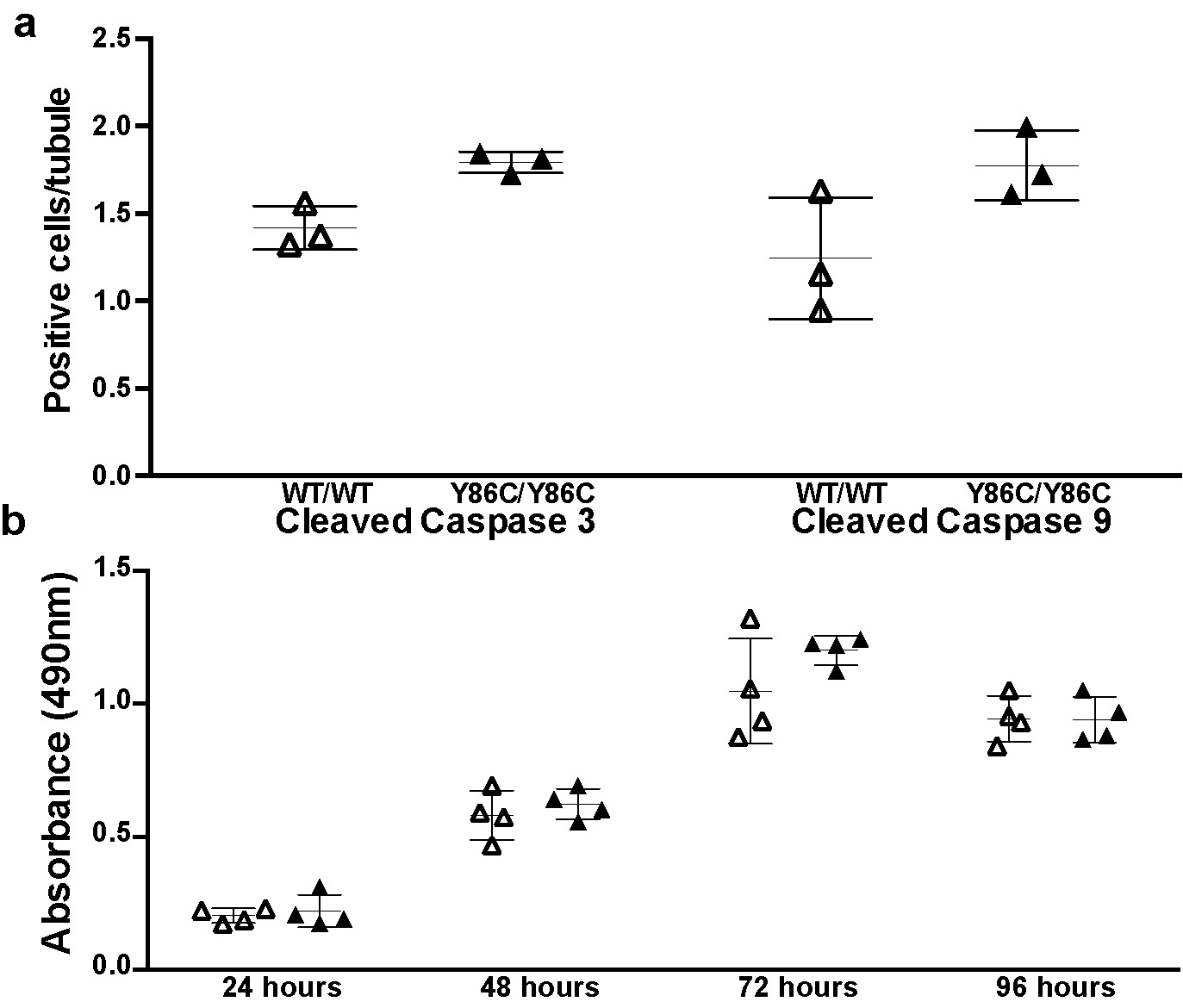

Supplement: S6 Fig — (a) Germ cell apoptosis in Katnal2Y86C/Y86C mice. The average number of germ cells per seminiferous tubules positive for either cleaved-caspase 3 or cleaved-caspase 9 showed no difference between Katnal2WT/WT and Katnal2Y86C/Y86C mice (n = 3/genotype). Lines represent mean ± SD. (b) KATNAL2 overexpression has no influence cell cycle progression. HEK293T cells stably transfected with a cumate inducible Katnal2 plasmid showed no difference in relative cell density using an MTS assay when cultured either in the absence (white triangles) or presence (black triangles) of cumate induction media at 24 hours, 48 hours, 72 hours and 96 hours after addition of media (n = 3/genotype). Lines represent mean ± SD. (TIF) [file pgen.1007078.s008.tif]

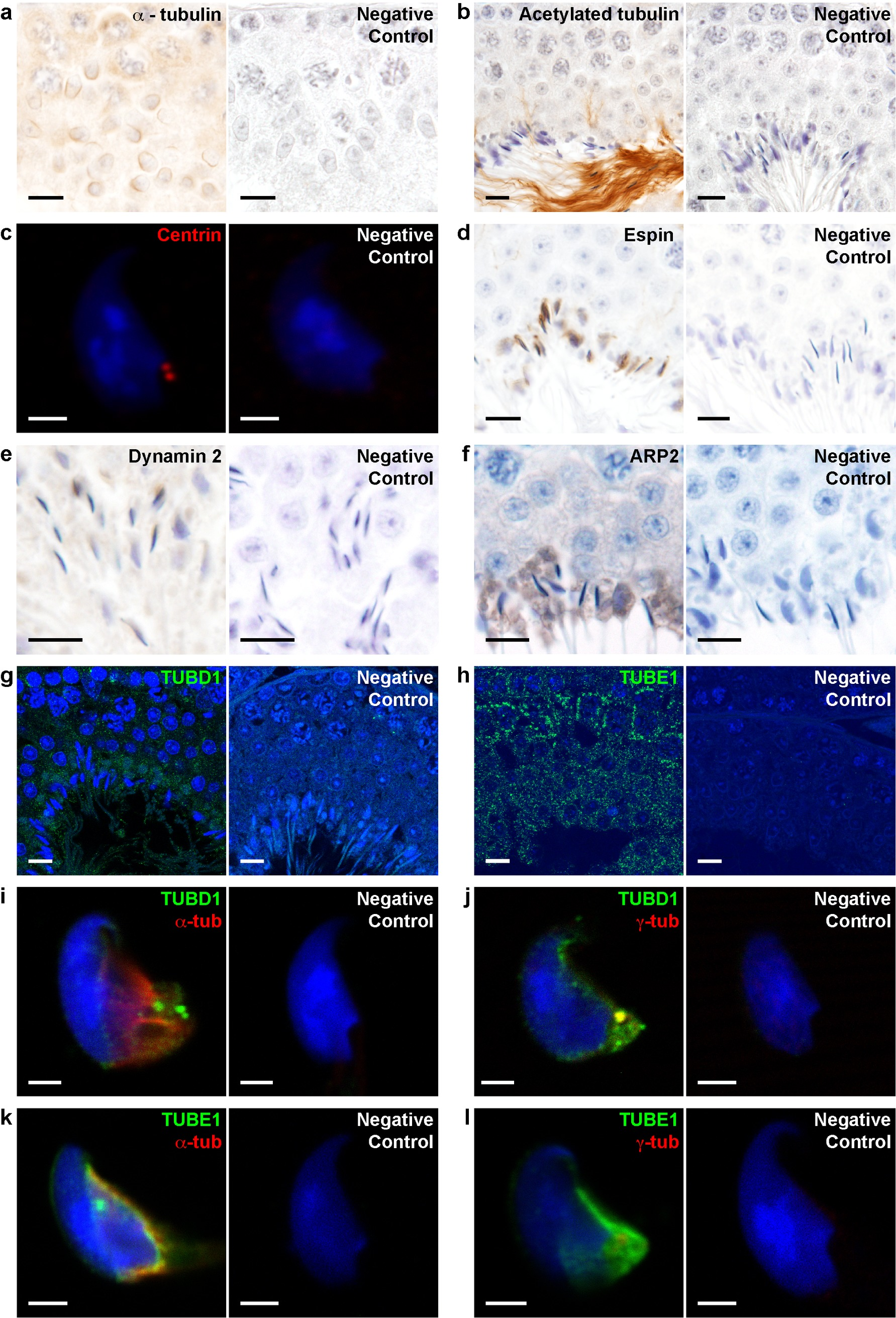

Supplement: S7 Fig — The specificity of immunolabelling as shown by the staining of parallel samples in the absence of primary antibody. α-tubulin (a) and acetylated tubulin (b) testis immunohistochemistry and corresponding primary antibody negative controls. (c) Centrin immunolabelling (red) on isolated germ cells and corresponding primary antibody negative control. Espin (d), dynamin-2 (e) and ARP2 (f) testis immunohistochemistry and corresponding primary antibody negative controls. TUBD1 (g) and TUBE1 (h) testis immunolabelling and corresponding primary antibody negative controls. TUBD1 (green) and α-tubulin (red) (i), TUBD1 (green) and γ-tubulin (red) (j), TUBE1 (green) and α-tubulin (red) (k), and TUBE1 (green) and γ-tubulin (red) (l) immunolabelling on isolated germ cells and corresponding primary antibody negative controls. In (a–b) and (d–f) nuclei are counterstained with haematoxylin. In (c) and (i–l) blue represents DNA as labeled by DAPI. In (g–h) blue represents DNA as labeled by TOPRO. In (a–b) and (d–h) scale bars = 10 μm and in (c) and (i–l) scale bars = 2 μm. (TIF) [file pgen.1007078.s009.tif]

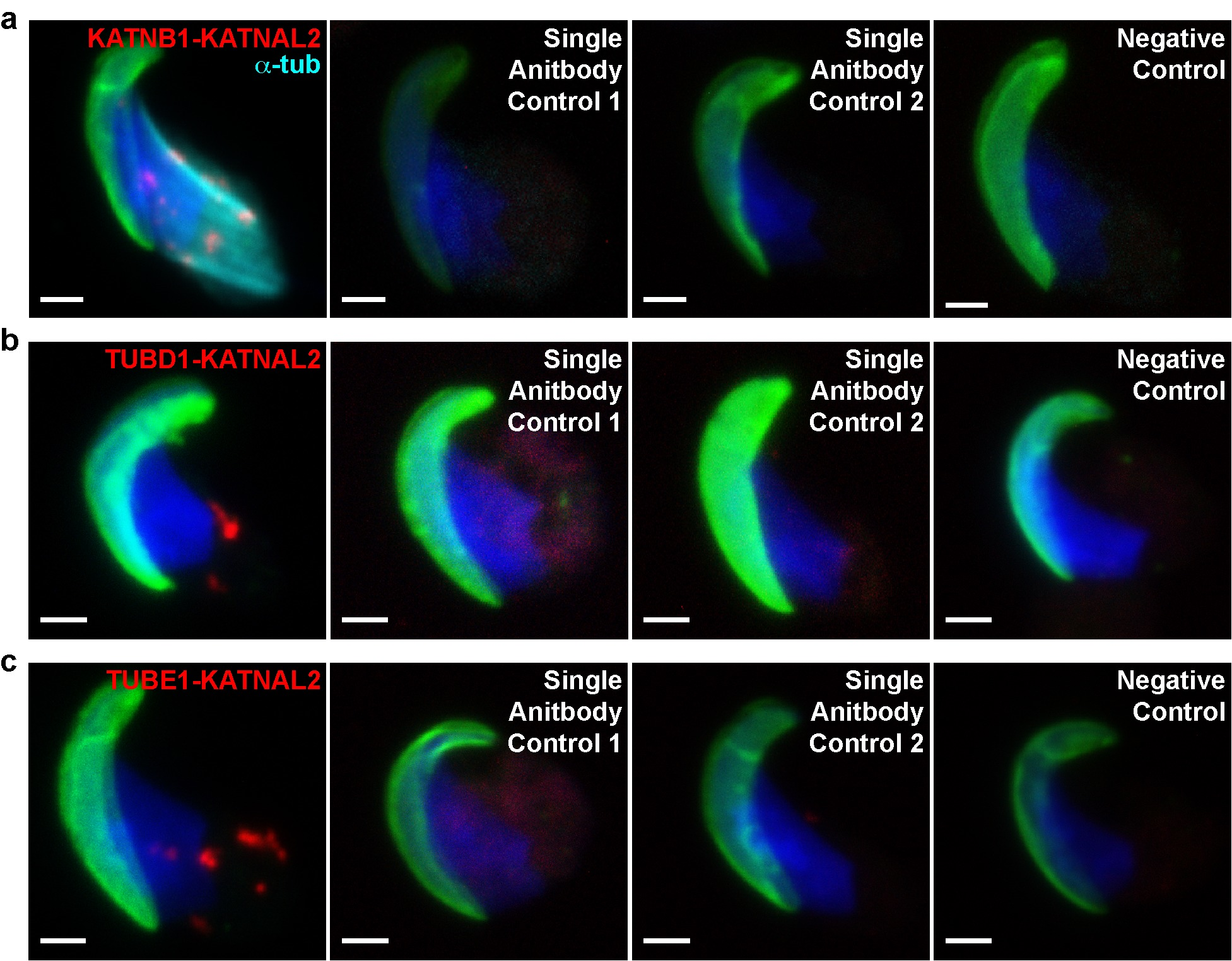

Supplement: S8 Fig — The specificity of the in situ proximity ligation assays as shown by the staining of parallel samples in the absence of either both or one of the primary antibodies. In situ proximity ligation assays using antibodies directed against KATNB1 and KATNAL2 (a), TUBD1 and KATNAL2 (b), and TUBE1 and KATNAL2 (c) in isolated Katnal2WT/WT spermatids. (a–c) Single antibody control 1: Assay was conducted with only KATNAL2 antibody. Single antibody control 2: Assay was conducted with only (a) KATNB1 antibody, (b) TUBD1 antibody and (c) TUBE1 antibody. (a–c) Negative control: Assay was conducted in the absence of all primary antibodies. (a) Cells were counterstained for α-tubulin (cyan) as a marker of microtubules. (a–c) Blue represents DNA as labeled by DAPI and green represents the acrosome as labeled by PNA. Scale bars = 2 μm. (TIF) [file pgen.1007078.s010.tif]
